# Regulatory role of the endocannabinoid system on glial cells toward cognitive function in Alzheimer’s disease: A systematic review and meta-analysis of animal studies

**DOI:** 10.3389/fphar.2023.1053680

**Published:** 2023-03-03

**Authors:** Mohd Amir Kamaruzzaman, Muhammad Hibatullah Romli, Razif Abas, Sharmili Vidyadaran, Mohamad Taufik Hidayat Baharuldin, Muhammad Luqman Nasaruddin, Vishnumukkala Thirupathirao, Sreenivasulu Sura, Kabul Warsito, Nurul Huda Mohd Nor, Muhammad Amsyar Azwaruddin, Mohammed Abdullah Alshawsh, Mohamad Aris Mohd Moklas

**Affiliations:** ^1^ Department of Anatomy, Faculty of Medicine, Universiti Kebangsaan Malaysia, Kuala Lumpur, Malaysia; ^2^ Department of Human Anatomy, Faculty of Medicine and Health Science, Universiti Putra Malaysia, Kuala Lumpur, Malaysia; ^3^ Department of Nursing and Rehabilitation, Faculty of Medicine and Health Science, Universiti Putra Malaysia, Kuala Lumpur, Malaysia; ^4^ Department of Pathology, Faculty of Medicine and Health Science, Universiti Putra Malaysia, Kuala Lumpur, Malaysia; ^5^ Department of Anatomy, Faculty of Medicine and Defence Health, Universiti Pertahanan Nasional Malaysia, Kuala Lumpur, Malaysia; ^6^ Department of Biochemistry, Faculty of Medicine, Universiti Kebangsaan Malaysia, Kuala Lumpur, Malaysia; ^7^ Department of Human Biology, International Medical University, Kuala Lumpur, Malaysia; ^8^ Department of Preclinical Sciences, Faculty of Medicine and Health Sciences, University Tunku Abdul Rahman, Kampar, Malaysia; ^9^ Department of Agrotechnology, Faculty of Science and Technology, University of Pembangunan Panca Budi, Medan, Indonesia; ^10^ Department of Pharmacology, Faculty of Medicine, Universiti Malaya, Kuala Lumpur, Malaysia; ^11^ Department of Paediatrics, School of Clinical Sciences, Faculty of Medicine, Nursing and Health Science, Monash University, Clayton, VIC, Australia

**Keywords:** Alzheimer’s disease, cognition, dementia, endocannabinoid, glial cell, microglia, astrocyte, systematic review

## Abstract

**Objective:** Over the last decade, researchers have sought to develop novel medications against dementia. One potential agent under investigation is cannabinoids. This review systematically appraised and meta-analyzed published pre-clinical research on the mechanism of endocannabinoid system modulation in glial cells and their effects on cognitive function in animal models of Alzheimer’s disease (AD).

**Methods:** A systematic review complying with PRISMA guidelines was conducted. Six databases were searched: EBSCOHost, Scopus, PubMed, CINAHL, Cochrane, and Web of Science, using the keywords AD, cannabinoid, glial cells, and cognition. The methodological quality of each selected pre-clinical study was evaluated using the SYRCLE risk of bias tool. A random-effects model was applied to analyze the data and calculate the effect size, while I^2^ and *p*-values were used to assess heterogeneity.

**Results:** The analysis included 26 original articles describing (1050 rodents) with AD-like symptoms. Rodents treated with cannabinoid agonists showed significant reductions in escape latency (standard mean difference [SMD] = −1.26; 95% confidence interval [CI]: −1.77 to −0.76, *p* < 0.00001) and ability to discriminate novel objects (SMD = 1.40; 95% CI: 1.04 to 1.76, *p* < 0.00001) compared to the control group. Furthermore, a significant decrease in Aβ plaques (SMD = −0.91; 95% CI: −1.55 to −0.27, *p* = 0.006) was observed in the endocannabinoid-treated group compared to the control group. Trends were observed toward neuroprotection, as represented by decreased levels of glial cell markers including glial fibrillary acid protein (SMD = −1.47; 95% CI: −2.56 to −0.38, *p* = 0.008) and Iba1 (SMD = −1.67; 95% CI: −2.56 to −0.79, *p* = 0.0002). Studies on the wild-type mice demonstrated significantly decreased levels of pro-inflammatory markers TNF-α, IL-1, and IL-6 (SMD = −2.28; 95% CI: −3.15 to −1.41, *p* = 0.00001). Despite the non-significant decrease in pro-inflammatory marker levels in transgenic mice (SMD = −0.47; 95% CI: −1.03 to 0.08, *p* = 0.09), the result favored the endocannabinoid-treated group over the control group.

**Conclusion:** The revised data suggested that endocannabinoid stimulation promotes cognitive function *via* modulation of glial cells by decreasing pro-inflammatory markers in AD-like rodent models. Thus, cannabinoid agents may be required to modulate the downstream chain of effect to enhance cognitive stability against concurrent neuroinflammation in AD. Population-based studies and well-designed clinical trials are required to characterize the acceptability and real-world effectiveness of cannabinoid agents.

**Systematic Review Registration:** [https://inplasy.com/inplasy-2022-8-0094/], identifier [Inplasy Protocol 3770].

## 1 Introduction

Alzheimer’s disease (AD) is a progressive neurological illness most commonly linked to memory loss and cognitive impairment (Liu & Li, 2019). However, other clinical manifestations are becoming more widely recognized. The presence of amyloid plaques and neurofibrillary tangles (NFTs) is currently essential for a pathological diagnosis (Mattson & Arumugam, 2018). AD is the major cause of dementia worldwide. Apart from a small percentage of cases attributed to familial genetic mutations, most AD cases do not have a clear underlying cause (Alzheimer’s Association Report, 2020). Proteinopathy (amyloid and tau) is a common feature in patients with AD and is typically associated with other age-related disorders, including cerebrovascular and Lewy body diseases (Deture & Dickson, 2019). Although cannabinoids have been rigorously studied in animal and clinical contexts in the field of AD, the cellular and biomolecular mechanisms and targets remain to be elucidated, challenging efforts to develop effective diagnostic tools and disease-modifying therapeutics (Deture & Dickson, 2019).

### 1.1 Cannabis ethnopharmacology

Since ancient times, Indian hemp, also known as cannabis sativa L. (*Cannabis sativa*), has been grown mostly in Central Asia (China and India) (Russo et al., 2008). It contains chemically active substances, including alkaloids, flavonoids, terpenoids, and cannabinoids (Andre et al., 2016). The most potent substances are terpenophenolic compounds, known as cannabinoids, which are primarily deposited in the female flowers' trichome cavity (Taura et al., 2007; Hartsel et al., 2016; Degenhardt et al., 2017). Numerous traditional medical applications of cannabis have already been established and are now accepted practices in medicine (Balant et al., 2021).

Phytocannabinoids, a naturally occurring, distinctive family of secondary metabolites, were believed to be responsible for the medicinal properties of cannabis. In 1964, Mechoulam and others first isolated and structurally elucidated the most abundant phytocannabinoid, a psychoactive ()-trans-9-tetrahydrocannabinol (THC) (Gaoni & Mechoulam, 1964). A non-psychoactive cannabidiol (CBD) was initially extracted in 1940 (Adams et al., 1939) and its full chemical structure was elucidated in 1963 (Mechoulam & Shvo, 1963). The finding of endogenous cannabinoids (endocannabinoids) in vertebrates as a result of the separation of phytocannabinoids from the cannabis plant has accelerated the growth of cannabis-related research (Turner et al., 2017).

Followers of Tantric Buddhism and Hinduism have utilized *C. sativa* flowers and resins in India and Tibet to aid in meditation and spirit communication (Schultes et al., 1992). The vegetal oils and proteins in *C. sativa* seeds were primarily employed by Chinese healers. According to Anwar et al. (2006), *C. sativa* seeds are high in linoleic acid, which doctors suggested using topically for inflammatory illnesses like eczema and psoriasis (Jeong et al., 2014). Historically, *C. sativa* was reportedly utilized by ancient Egyptian ladies to ease pain and elevate their mood, according to Diodorus Siculus (about 60 B.C.) (Bonini et al., 2018). Pliny the Elder, a Roman historian, remarked on the use of *C. sativa* roots for pain relief (Ryz et al., 2017). *C. sativa* was used in nineteenth-century English medicine as an analgesic, anti-inflammatory, anti-emetic, and anti-convulsant (Bonini et al., 2018; Balant et al., 2021).

### 1.2 Endocannabinoid system and cognitive abilities

The endocannabinoid system (ECS) consists of peptide (hemopressin derivatives) and lipid endocannabinoid (eCB) mediators, cannabinoid receptors, membrane transporters, and metabolic enzymes (Augusto et al., 2021). Within the central nervous system (CNS), the ECS regulates synaptic transmission, synaptic plasticity, and cytokine release, in addition to playing a neuroprotective role against neuronal injury (Cristino et al., 2019). The two primary receptors that make up the ECS are cannabinoid receptor type 1 (CB1R) and cannabinoid receptor type 2 (CB2R). As the most abundant G protein-coupled receptor (GPCR) in the brain, CB1R is found in the anterior cingulate cortex, prefrontal cortex, striatum, and hippocampus (Morena & Campolongo, 2014; Di Marzo et al., 2015). Another GPCR, CB2R is expressed mostly in the cellular immune system (natural killer cells, B cells, macrophages, and activated microglia) (Zou & Kumar, 2018) and recent immunostaining and western blotting studies have identified its weak expression in healthy neural tissue of rats, mice, ferrets, and human (Stempel et al., 2016; Zou & Kumar, 2018; Komorowska-Müller & Schmöle, 2021). AEA and 2-AG are the two most studied endocannabinoids, with the former a partial agonist at CB1R and CB2R and the latter a full agonist at these two sites when expressed at concentrations 200 times greater than those found in the CNS (Bajaj et al., 2021). Besides CB1R and CB2R, the ECS has several other receptors, including nuclear receptors and transient receptor potential channel ionotropic receptors (Marcu & Schechter, 2016). Derived from lipid membrane components that go through on-demand synthesis, endocannabinoids inhibit the retrograde of neurotransmitter release from adjacent neurons *via* CB1R-mediated signaling. The degradative enzymes also have a significant role in regulating endocannabinoid activity efficiently, with 2-AG predominantly degraded by monoacylglycerol lipase (MAGL) and fatty acid amide hydrolase (FAAH) playing the same role for AEA (Tanaka et al., 2020).

Accumulating evidence suggests the benefits of endocannabinoids on emotion and cognitive abilities (Morena & Campolongo, 2014; Kruk-Slomka et al., 2017). Infusions of AM251 and rimonabant, CB1R antagonists/inverse agonists, into the hippocampus region dramatically diminished memory updating, limited behavioral flexibility, and promoted the forgetting of fearful memories in Wistar rats (Lunardi et al., 2020). Conversely, the microinjection of arachidonylcyclopropylamide (1–4 ng/rat), a CB1R agonist, into the rat’s basolateral amygdala ameliorated a scopolamine-induced memory consolidation deficit (Nedaei et al., 2016). Additionally, endocannabinoid signaling engaged dorsal striatum glucocorticoids by promoting memory consolidation in Wistar rats, as evidenced by enhanced retention avoidance following inhibitory avoidance training (Siller-Pérez et al., 2019). In the Morris water maze test, the CB agonist WIN55,212–2 affected spatial memory acquisition but not consolidation. Moreover, endocannabinoid signaling also facilitated reward-based motor sequence learning, which was disrupted in CB1 and diacylglycerol lipase-a (DGLa) knockout mice (Tanigami et al., 2019).

### 1.3 Evidence of cognition-associated glial cells

Major advancements in the understanding of microglia and astrocytes have been made in recent years. Glial cells are activated or reactivated in a variety of pathogenic states, including stroke, trauma, tumor growth, and neurodegenerative disorders. Neuroinflammation can cause two distinct forms of reactive astrocytes, A1 and A2, which correspond to the M1/M2 phenotypes of microglia and macrophage classification (Liddelow & Barres, 2017).

Microglia play essential roles in CNS development, immunological surveillance, and maintenance of neuronal function (Maurya et al., 2021). Microglia are activated and acquire different transcriptome profiles in neurological diseases, including disease-associated microglia (DAM), which have been linked to neurodegenerative disorders (Deczkowska et al., 2018). Microglia also have a detrimental role in learning and memory capabilities under inflammatory and disease conditions. Minett et al. reported that the presence of dementia was positively associated with microglial activation markers (CD68 and CD64), with an inverse relationship with Iba1, a pan microglial marker (Minett et al., 2016), as indicated by a loss of microglial motility necessary to support neurons (Franco-Bocanegra et al., 2019). Persistent inflammatory pain elevated microglial activity in the dentate gyrus (DG) and cornu ammonis (CA1 subfield) regions, as evidenced by cellular changes that impaired spatial learning and memory abilities (Mohammadi et al., 2020). Transformation of the microglial phenotype from an active to an alternate state is important to maintain neuronal stability. CD200R, a receptor expressed by microglial cells, interacts with CD200 molecules released by neurons, astrocytes, and oligodendrocytes to enhance phagocytosis, providing a potential neuroprotective effect. CD200 suppression by hippocampus induction *via* AAV injection revealed severe impairment of synaptic and cognitive function (Feng et al., 2019).

Astrocytes are the most abundant and largest type of glial cells in the CNS, where they play a critical role in synaptic transmission and plasticity, neuroprotection, and maintenance of CNS homeostasis (Kasatkina et al., 2021). Astrocytes are also essential contributors to information processing and cognitive behavior. In the context of AD, astrocytes have received less attention than microglia or neurons. Fortunately, recent technologically advanced tools such as optogenetics, scRNAseq, in vivo imaging, and the growing use of animal and cell models (iPSCs), have illuminated the functions of astrocytes in normal and pathological conditions (Escartin et al., 2021; Sanchez-Varo et al., 2022). In *in vitro* and *in vivo* experiments, Lee et al. (2014)demonstrated that astrocytes are necessary for novel object recognition behavior and functional gamma oscillations maintenance. Moreover, evidence supports the role of astrocytes in the regulation of neuronal oscillations and cognitive flexibility *via* the Ca^2+^ binding protein S100β at the medial prefrontal cortex (mPFC) of rats (Brockett et al., 2018). Regarding the entorhinal cortex-dentate gyrus circuit, a review by Di Castro and Volterra showed that increased TNFα levels during infection/inflammation processes led to uncontrolled astrocyte glutamate release, altering perforant path (PP) excitatory projections onto dentate granule cells (GC). The disruption of this PP-GC synapse processing ultimately impaired contextual memory performance (Di Castro & Volterra, 2022). Zhang et al. (2020) demonstrated that IL-10, an anti-inflammatory cytokine, is essential in the modulation of A1 astrocyte activation, learning, memory dysfunction, and depressive-like behavior.

### 1.4 Endocannabinoid-glial cell relationship

Microglial cell exposure to cannabinoids may result in the beneficial promotion of neurotrophic capabilities (Saijo & Glass, 2011). This is observed particularly when homeostatic microglia synthesize endogenous cannabinoids (AEA and 2-AG) along with the low expression of cannabinoid receptors (CB1R and CB2R). Microglia produce more endocannabinoids, which upregulate CB2R expression when activated, promoting a protective microglial phenotype by increasing the production of the neuroprotective molecules while decreasing the production of pro-inflammatory components (Komorowska-Müller & Schmöle, 2021). Additionally, microglial cells play a critical role in synaptic stripping, which occurs when synapses are removed from damaged neurons (Kettenmann et al., 2013). As the constant sensors of microenvironment changes in the CNS and restorer of tissue homeostasis, microglia not only serve as the primary immune cells of the CNS but also regulate the innate immune functions of astrocytes. The modulation of astrocyte function by endocannabinoid signaling is thought to be regulated by astrocyte Ca2+ mobilization by CB1R, which occurs throughout the rodent brain as well as in cortical and hippocampal human tissue (Bernal-Chico et al., 2022). Both microglia and astrocytes establish autocrine feedback and bidirectional communication for a tight reciprocal modulation upon CNS insult or injury by releasing a range of signaling molecules (Jha et al., 2019). Hence, this review critically discusses and summarizes the glial-endocannabinoid system effects on AD in animal models.

### 1.5 Research aims

The research question was generated based on the PICO model of research question as follows. In AD-like animal models, P) would modulation of the endocannabinoid system influence glial cells I) compared to control C) to improve cognitive function O)? As there is growing data regarding the effects of endocannabinoid and glial cells on cognition, the present study aimed to provide a detailed systematic literature review of existing animal research examining the effects of endocannabinoid modulation of glial cells and its mechanism on the cognitive domains relevant to AD. The research papers were selected in this review to meet the following objectives: 1) to evaluate the potential mechanisms of endocannabinoid mediated by glial cells on cognitive measurement in AD-like animal models; 2) to determine the amelioration of neuro-inflammatory and other relevant pathological markers based on the effects in endocannabinoid-based pre-clinical models of cognitive impairment; and 3) to compare the effects of cannabinoid administration on cognitive function and make recommendations for future research.

This systematic review conducted a meta-analysis of studies performed on rodent models of AD to provide a comprehensive evaluation and understanding of the efficacy of the endocannabinoid system in modulating glial cells in the context of cognitive function.

## 2 Materials and methods

The Preferred Reporting Items for Systematic Review and Meta-Analyses (PRISMA) criteria were used to conduct this systematic review. The protocol for this systematic review was registered in the INPLASY database (registration no INPLASY202280094). Only studies on glial cell alterations regulated by endocannabinoids were included in this review, along with behavioral evaluations following any quantity of cannabinoid agonist treatment or cannabinoid receptor knockout in mice or rats. For the meta-analysis, data that reported similar or related outcomes under similar or related experimental circumstances were pooled to calculate the final effect size. Other studies were qualitatively synthesized in this systematic review but were eliminated from the meta-analysis because they described different measurements or distinct sets of experiments.

### 2.1 Eligibility criteria and screening

Studies comparing the effects of endocannabinoid modulation on glial cells and related to behavior evaluation in rodents (mice and rats of either sex) were considered. The search was restricted to English-language articles that provided proof of experimental work in rodents. No consideration was given to the number of animals used in the experiment, transgenic or wild type, or the length of time the animals were exposed to the treatment. Studies with insufficient data were excluded from the meta-analysis. Results that allowed for the creation of pooled data were selected for inclusion in the meta-analysis.

The articles were initially screened based on their titles and abstracts, and any irrelevant studies were excluded. Two reviewers separately examined the abstracts to identify articles that fulfilled the inclusion criteria. Any disagreement was resolved by a discussion with a third reviewer. The remaining items were reviewed by reading the entire texts. Articles that were unrelated or that did not have the entire text available were excluded. The eligibility criteria were established based on the defined criteria for study inclusion and exclusion.

#### 2.1.1 Inclusion and exclusion criteria

The criteria for the selection of articles were based on the Population, Intervention, Comparison, and Outcomes (PICO) model as a framework to establish the inclusion criteria. The four main identification components were: 1) AD animal model; 2) endocannabinoid system (ligand, receptors, enzymes, exogenous cannabinoid, etc.); 3) glial cells (microglia, astrocytes, oligodendrocytes); and 4) cognitive outcome (maze/NOR, etc.). The inclusion and exclusion criteria are summarized in Table 1.

**TABLE 1:** Inclusion and exclusion criteria based on PICO model.

Inclusion criteria	Exclusion criteria
• Laboratory rodents of any species, age, sex, or weight-producing Alzheimer’s disease (AD) models. Any kind of induction in which the design was dedicated to AD as a primary model	• Non-animal studies
• Any comparison between endocannabinoid modulation and the control group. A placebo, such as physiological saline or some similar substance, included in the control group. No constraint on drug dosage, route of administration, or length of therapy	• Studies without AD models in animals. An induction not dedicated to the AD model as a primary model or if the model was non-specific/broad such as neurodegenerative/neuroinflammation
• Primary outcomes including measurements from cognitive tests. Secondary outcomes of glial cell immunoreaction/glial cell mechanism changes by any mean of measurement	• No control group
• Pathological changes were assessed and not restricted to amyloid beta (Aβ), which is the precipitated protein and the component of amyloid plaque found in the brain of patients with AD	• Studies lacking cognition results as a primary outcome
• Original experimental studies measuring the efficacy of endocannabinoid stimulation in AD animal models	• Studies lacking glial cell results as a secondary outcome
	• Other exclusion criteria include the duplication of references, review articles, lack of full text, and literature with incorrect or incomplete data

### 2.2 Data sources and search strategy

To identify studies examining endocannabinoid neuroprotective properties in AD models of rodents, a thorough literature search was conducted. The pertinent publications were gathered from six credible databases: five from the EBSCOhost platform (**Academic Search** Complete, CINAHL Plus with Full Text, Cochrane Central Register of Controlled Trials, MEDLINE Complete, and the Psychology and Behavioral Sciences Collection). Additionally, Scopus was also searched. The keywords used for the literature search were as follows.

i) “Alzheimer* disease” OR “dementia” OR “mental disorder” OR “mental deterioration” OR “neurodegenerati*” OR “neuroinflammat*”
ii) “endocannabinoid*” OR “endocannabinoid system*” OR “cannabinoid receptor*” OR “cannabinoid agonist*” OR “cannabinoid ligand*” OR “cannabinoid*” OR “cannabis” OR “cannabis sativa” OR “cannabidiol” OR “cbd” OR “tetrahydrocannabinol” OR “thc”
iii) “microglia*” OR “autophag*” OR “microglia* activation” OR “microglia* stimulation” OR “microglia* function” OR “microglia* polarization” OR “microglia* propert*” OR “glia*” OR “gli*” OR “immune cell*” OR “immunomodulat*” OR “neuroprotect*” OR “astrocyte*” OR “astrogli*” OR “oligodendrocyte*” OR “ependymal cell*”
iv) “cogniti*” OR “intelligence*” OR “intellectual*” OR “executive function*” OR “think*” OR “learn*” OR “memor*” OR “judge*” OR “knowledge” OR “mind” OR “thought” OR “behavio*”

Only studies concerning animal models were selected for further consideration. All the articles identified during the search were exported to Mendeley (Mendeley Ltd. ^©^ 2008—2020 version 1.19.8) and duplicate records were removed.

### 2.3 Critical appraisal and methodological quality assessment

Bias is a divergence from the truth in outcomes or inferences that can lead to systematic errors. The risks of bias among the included articles were assessed using a checklist developed by the Systematic Review Centre for Laboratory Animal Experimentation (SYRCLE) and based on the Cochrane Collaboration RoB Tool (Hooijmans et al., 2014). The checklist contains ten items within ten main domains: sequence generation, allocation concealment, baseline characteristics, random outcome assessment, random housing, blinding of the investigator, blinding of outcome evaluators, selective outcome reporting, incomplete outcome data, and other sources of bias. For the judgment of bias, the answer was either “Yes” to indicate a low risk of bias, “No” to indicate a high risk of bias, or “NC” to indicate an unclear level of bias due to inadequate information.

Evidence across studies was assessed for quality according to the Grades of Recommendation, Assessment, Development, and Evaluation (GRADE) Working Group (Schünemann et al., 2008). This evaluation considered the risk of bias within individual studies, the precision of effect estimates, heterogeneity, the directness of the evidence, and the risk of publication bias (Higgins et al., 2011).

The primary outcomes obtained in the selected articles described the presentation of general cognitive function (memory, learning, orientation, and attention) and the modifications observed as a result of endocannabinoid modulation methods in AD animal models. Moreover, secondary outcomes representing the animal’s biochemical characteristics and histological analysis were also observed. These included glial cell and inflammatory markers, amyloid burden, oxidative stress markers, synaptic plasticity, enzymatic levels, and cellular apoptosis.

### 2.4 Data synthesis and analysis

**FIGURE 1:**
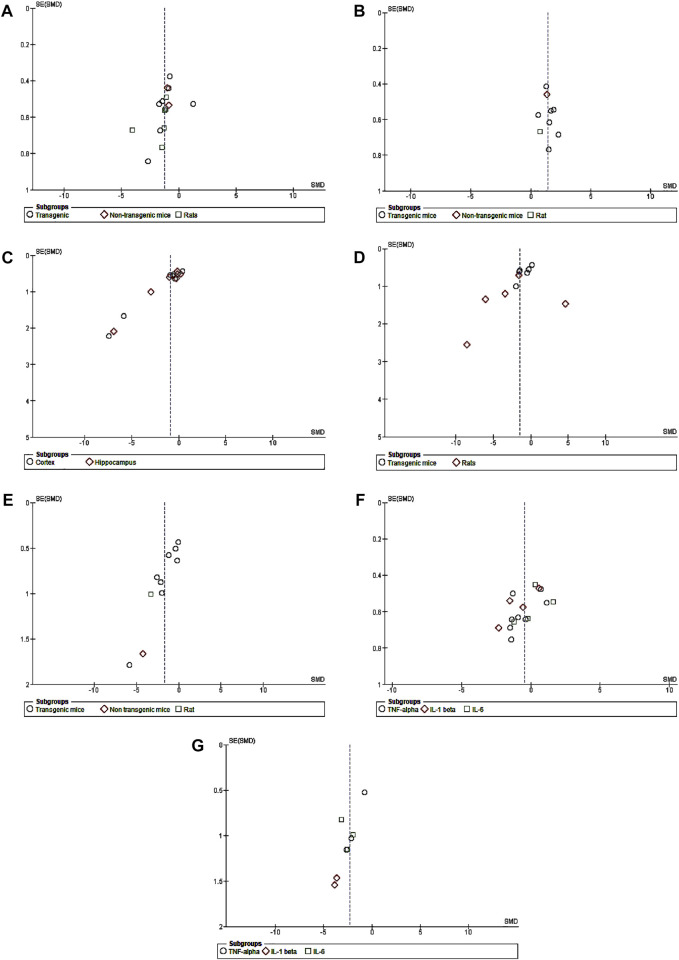
Funnel plots of publication bias. **(A)** CB treatment *versus* model for escape latency. **(B)** CB treatment *versus* model for recognition index. **(C)** CB treatment *versus* model for amyloid plaque. **(D)** CB treatment *versus* model for GFAP. **(E)** CB treatment *versus* model for Iba1. **(F)** CB treatment *versus* model for pro-inflammatory cytokines in transgenic mice. **(G)** CB treatment *versus* model for pro-inflammatory cytokines of non-transgenic mice.

The meta-analysis was performed using RevMan version 5.4 (The Nordic Cochrane Centre, The Cochrane Collaboration, Copenhagen, Denmark). The results consisted of several outcome variables including the number of right quadrant/range crossings during Morris water maze tests, levels of glial cell and inflammatory markers, and brain amyloid beta (Aβ), which were determined using a random-effects model to account for the possibility of heterogeneity because identical outcome variables were used to analyze various AD animal models in each study. The standard mean difference (SMD) and 95 percent confidence intervals (CIs) were calculated using a random-effects model for each study. To determine heterogeneity, the Q-statistic test was utilized. *P* <0.05 indicated heterogeneity among the studies. I^2^ values were used to measure the heterogeneity. When *p* < 0.05, heterogeneity was considered to be present. I^2^ = 0%, 0 < I^2^ ≤ 25%, 25% < I^2^ ≤ 75%, and I^2^ > 75% indicated no, mild, moderate, and high heterogeneity, respectively. By removing the included data one at a time, a sensitivity analysis was carried out to determine if these modifications altered the estimated cumulative result effect size when there was substantial heterogeneity in the data (Bown and Sutton, 2010). Data were extracted from plots/images using WebPlotDigitizer version 4.5 (Cramond et al., 2019). A meta-analysis was then performed on the extracted data using RevMan 5.4. The presence of publication bias was investigated using a graphical funnel plot (Figure 1).

## 3 Results

### 3.1 Study selection and data extraction

**FIGURE 2:**
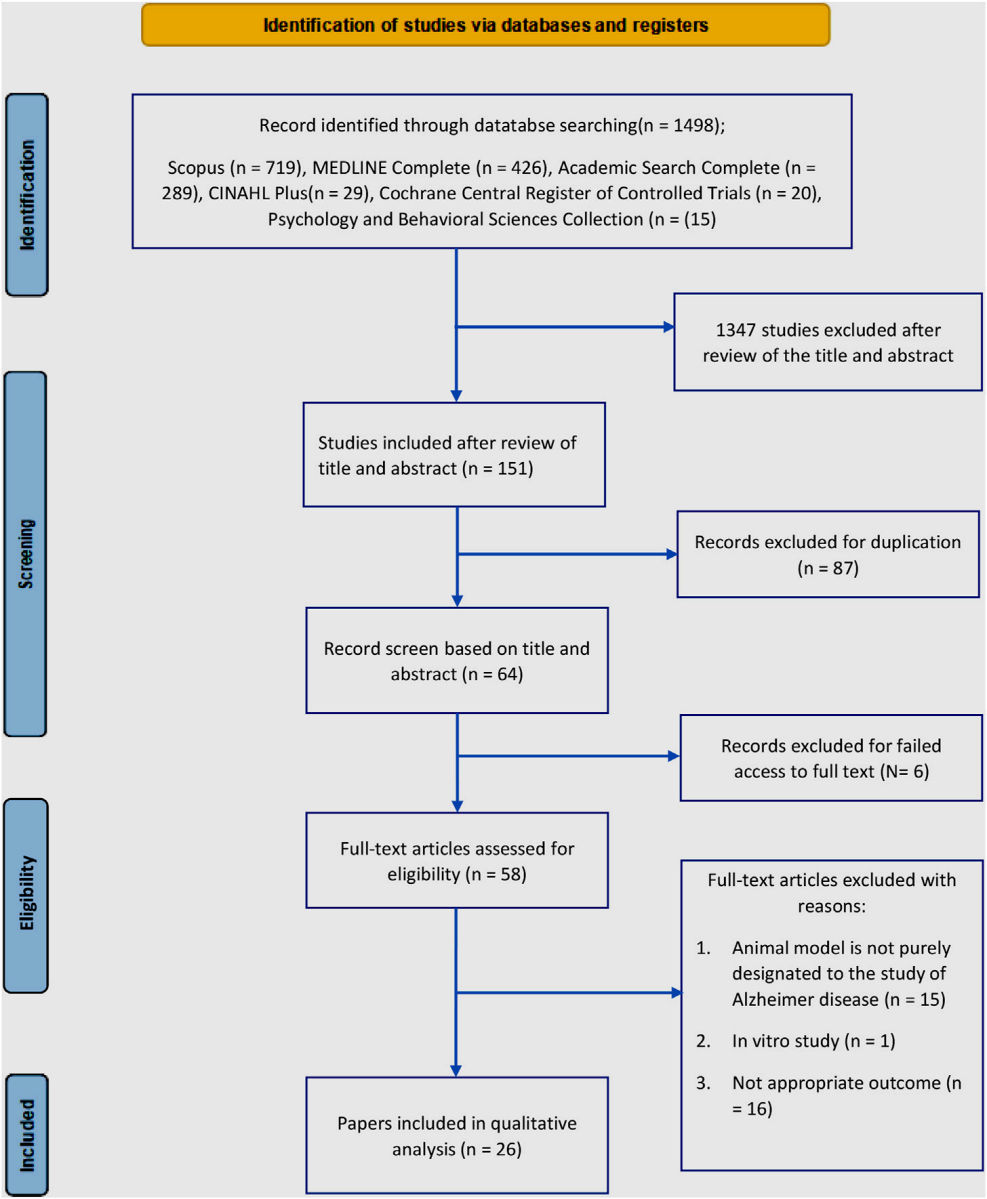
lowchart of the literature search.

The databases were searched from January to July 2022, and a total of 1,498 records were identified. Subsequently, 1,347 irrelevant articles were excluded (most did not use AD animal models or did not report the association between endocannabinoid-glial cells-behavior and cognition), leaving 151 records. Further assessment revealed that 87 articles were duplicates, which were removed. A further 17 records were excluded due to either absence or inability to access the full texts (Supplementary Table S1). After reviewing the full texts of the remaining 47 records, 26 studies met the inclusion criteria. Additionally, more than half of the articles included were published within the last 10 years (i.e., 2012–2022). The relevance of the inclusion criteria for this review exists since 2005, when the first included article was published. Thereafter, articles on the involvement of the endocannabinoid system in modulating glial cells in AD-like rodent models indicate an ongoing research interest. Figure 2 illustrates the PRISMA flow diagram of the search results.

### 3.2 Study characteristics

The systematic literature search found no human clinical evidence of the effects of cannabinoid treatment on cognition in AD based on the observation of glial endocannabinoid signaling. This may be because the symptoms of AD typically appear in humans at the moderate to advanced stages of disease. Moreover, the pathological evidence can only be observed in post-mortem harvesting of brain tissue. Mammalian rodent models such as mice and rats that are commonly used for AD are more receptive to transgenic procedures, easier to maintain, less expensive, and have shorter lifespans than invertebrates and non-mammalian vertebrates (NHP). As such, utilizing mouse models with human neurodegeneration may be more appropriate and realistic (Leung & Jia, 2016). Furthermore, the cognitive capacities in rodent models have been extensively studied and faithfully mimic the neurodegeneration and accompanying cognitive and behavioral abnormalities as those seen in humans (Webster et al., 2014). Rats and mice are generally models that best suit this research area. The identified rat/mouse studies in the present review fulfilled the criteria for the association of endocannabinoid-glial cell-cognitive function.

Exploring the pathophysiology and determining the therapeutic efficacy of AD requires a reliable experimental model. Of the 26 included articles, most toxin-induced AD models did not accurately reflect acute toxicity-induced neurodegeneration. Some did not create NFTs or Aβ deposition linked to AD. The transgenic mouse models with specific transgenes to replicate AD pathology appeared to be a more suitable tool for therapeutic discovery. To provide objective support and unbiased evidence for the continued application of cannabinoid-based agents as candidate drugs in the clinical treatment of AD, this study thoroughly examined and systematically analyzed the neuroprotective effects of endocannabinoid-mediated glial cells in various rodent models of AD. A summary of the key characteristics of the included studies is provided in Table 2.

**TABLE 2:** Characteristics of the included studies.

No	Study	Animal model construction	Comparators	eCB modulated method/treatment	Glial cell immuno-reaction, activation/M1/M2 phenotype	Other relevant key findings	Behavioral test	Cognitive domain	Pathology	Antagonist test	References
1	Improved cognition impairment by activating cannabinoid receptor type 2: Modulating CREB/BDNF expression and impeding TLR-4/NFκB/M1 microglia signaling pathway in D-galactose-injected ovariectomized rats	Injection of D-galactose (150 mg/kg, i.p.) to induce AD-like features in bilaterally ovariectomized female rats (OVC/D-gal rats) for 8-week	1. Sham-operated group	AM1241, a CB2R-agonist (3 and 6 mg/kg), was injected intraperitoneally starting from the 6th week until 8th week	GFAP ↓	Pro-infl: NF-κB p65, TNF-α, IL-6 and IL-12 ↓	Novel object recognition (NOR), Morris water maze	Discrimination index ↑; Preference index ↑; Escape latency ↓; time spent in target quadrant ↑	Aβ plaque ↓	-	Abd El-Rahman & Fayed (2022)
			2. OVC/D-gal group		CD68 ↓	Anti-infl: IL-4 and IL-10 ↑					
			3. OVC/D-gal + AM1241 groups (3 mg)			TLR-4 ↓; Myd88 ↓					
			4. OVC/D-gal + AM1241 groups (6 mg)			BDNF ↑; CREB ↑; Caspase 3					
			(n = 20) per group			OVC/D-gal + AM1241: neuronal cell degeneration ↓					
2	Activation of GPR55 attenuates cognitive impairment, oxidative stress, neuroinflammation, and synaptic dysfunction in a streptozotocin-induced Alzheimer’s mouse model	Male ICR mice (6 weeks old) underwent stereotaxic administration of STZ by ICV	1. Veh + Veh	PBS (3 μL) with or without O-1602 infused by ICV. (acute administration)	Iba1 ↓ (DG & frontal cortex)	Hippocampus & frontal cortex	Novel object recognition (NOR); Morris water maze	Discrimination index ↑; Escape latency ↓; time spent in target quadrant ↑	Aβ1–42 levels ↓	-	Xiang et al. (2022)
			2. STZ + Veh			TNF-α, IL-1β, and IL-6 ↓					
			3. STZ + O-1602 2.0 μg/mouse			AChE activity ↓; BACE1 ↓					
			4. STZ + O-1602 4.0 μg/mouse			GPR55 expression ↑					
			5. (n = 12) per group			MDA ↓; SOD ↑					
3	Cannabinoid receptor CB2 ablation protects against TAU-induced neurodegeneration	7 and 12-month-old male transgenic mice overexpressing hTAUP301S Wild type (Cnr2+/+) and CB2-knockout (Cnr2−/−) induced by hTAU P301L Post-mortem of patients with AD patients	1. Control	Viral vectors of serotype 6, which express hTAUP301L under control of the human synapsin 1 gene promoter (AAV-hTAUP301L), were injected in the right hippocampus (ipsilateral side) into (Cnr2+/+) and (Cnr2−/−) mice	hTau overexpression: GFAP ↑; amoeboid-reactive form (M1)	hTau overexpression: Cnr2 ↑; CB2 expression ↑; NF-κB, Il-1β ↑; AEA level ↑; FAAH expression ↓	Novel object recognition (NOR)	Cnr2+/+ mice with AAV-hTAUP301L: recognition memory ↓	-	-	Galán-Ganga et al. (2021)
			2. hTAUP301S		hTAUP301L*Cnr2−/−: GFAP no changes	AAV-hTAUP301L*Cnr2−/−: Bdnf expression ↑					
			3. TAU-KO			AAV-hTAUP301L*Cnr2−/−: loss of part of the granular cell layer ↓					
			4. AAV-hTAUP301L*Cnr2+/+			Postmortem					
			5. AAV-hTAUP301L*Cnr2−/−			CB2, mRNA levels of CNR1 and CNR2 ↑ in neurofibrillary tangle TAU positive neurons from AD patients					
			(n = 8–15) per group								
4	WIN55,212-2 attenuates cognitive impairments in AlCl3 + D-galactose-induced Alzheimer’s disease	Albino Wistar rats (male), 2–3 months old (200–350g); induction with daily AlCl3 (orally) + D-gal (i.p)	1) Control	Administration of WIN55,212-2 (WIN) (0.5, 1 and 2 mg/kg/day), from weeks 8–11	GFAP ↑	Nestin level ↑ SOD ↑	Morris water maze	Escape latency ↓; distance coverage ↑; time spent in target quadrant ↑	-	-	Mahdi et al. (2021)
			2) Model								
			3) Donepezil								
			4) WIN0.5 mg/kg								
			5) WIN1mg/kg								
			6) WIN2mg/kg								
			(n = 6) each group								
5	CB2 cannabinoid receptor agonist ameliorates novel object recognition but not spatial memory in transgenic APP/PS1 mice	Transgenic APP/PS1 mice (male) (APPswe and PS1dE9)	1) WT + VEH	Each morning for 8 weeks, animals were injected with VEH or JWH-015 at a dose of 0.5 mg/kg intra-peritoneally in a volume of 20 mL/kg (purchased)	Iba1 expression ↓ (cortex); M2 marker Ym1/2 ↑	Basal dendritic length, branching points, and spine density (cortex) ↑ In cortex: M1 markers (IL-6, TNF-α and iNOS) ↓	Novel object recognition test (NOR); Morris water maze test (MWM)	Recognition index (RI) ↑	Aβ plaque number: no difference	-	C. Li et al. (2019)
			2) APP/PS1 + VEH								
			3) APP/PS1 + JWH-015								
			(n = 5) each group								
6	Acute activation of the CB1 receptor in the hippocampus decreases neurotoxicity and prevents spatial memory impairment in rats lesioned with β-amyloid 25-35	Adult (male) Wistar rats (260–300g). 1 μL of Aβ (35 -25) (100 μM) + 1 μL of PBS, both into the CA1 subfield of the hippocampus via stereotaxic administration	1) Vehicle	Animals were placed in a stereotaxic apparatus for the administration of 1 μL of ACEA (1 μM) (purchased) (acute administration)	GFAP ↓ (Hippocampus) GFAP ↓; Iba1 ↓ (Dentate gyrus)	iNOS expression ↓; NO ● ↓; fluoro -Jade B stain ↓	eight -arm radial maze	correct responses ↑; reference errors ↓; latency to the third correct response ↓	-	AM -251	Patricio-Martínez et al. (2019)
			2) Aβ(35 -25)								
			3) ACEA								
			4) AM-251								
			5) Aβ(25–35)								
			6) ACEA + Aβ(25–35)								
			7) AM-251+Aβ(25–35)								
			8) AM-251 + ACEA + Aβ(25–35)								
			(n = 12) each group								
7	Alleviation of neuropathology by Inhibition of monoacylglycerol lipase in APP transgenic Mice lacking CB2 receptors	5XFAD transgenic mice (female)	1. WT-CB2KO-Veh	TG-CB2-KO mice were treated with JZL184 (12 mg/kg, i.p.) three times per week starting at 4 months of age for 8 weeks (MAGL inhibitor) (provided)	GFAP ↓	Aβ plaques ↓ Cortex & hippocampus: BACE1 ↓; Aβ42; CTFβ/α; FJC-positive ↓ GluA1, GluA2, PSD95 ↑	Morris water maze	Escape latency ↓; target quadrant time ↑ thus learning acquisition and memory retention ↑	Aβ plaque ↓	-	J. Zhang and Chen. (2018)
			2. TG-CB2KO-Veh								
			3. TG-CB2KO-JZL								
			(n = 10) each group								
8	Cannabinoid receptor 2-deficiency ameliorates disease symptoms in a mouse model with Alzheimer’s disease-like pathology	APP/PS1 mice	1. WT	APP/PS1 mice were crossed with tau mice. APP/PS1*CB2þ/mice were then crossed with CB2−/− mice to become APP/PS1*CB2−/− mice	APP/PS1*CB2−/−: microglia ramification index, plaque shape index ↓	APP/PS1*CB2−/−: Aβ plaques in cortex & hippocampus: ↓ APP/PS1*CB2−/−: adam17/ide ↑; ager ↓ APP/PS1*CB2−/−: NeuN+ & Parv+ in cortex ↑	Morris water maze	APP/PS1*CB2−/−: escape latency @ D3/D5 ↓	Aβ plaque shape index ↓	-	Schmöle et al. (2018)
			2. CB2−/−								
			3. APP/PS1								
			4. APP/PS1*CB2−/−								
			(n = 8–16) each group								
9	Role of interleukin 1-beta in the inflammatory response in a fatty acid amide hydrolase-knockout mouse model of Alzheimer’s disease	5xFAD mice (male/female)	1. WT	5xFAD mice were back crossed with FAAH−/− mice to obtain the 5xFAD/FAAH−/−. The animals were treated with anti-inflammatory minocycline (Mino)	5xFAD/FAAH−/−: M1/M2 ratio ↑; microglia number ↓	5xFAD/FAAH−/−: IL-1β TNFα ↑; IL10 and IL4 ↓ 5xFAD/FAAH−/−/Mino: IL-1β mRNA ↓ 5xFAD/FAAH−/−/Mino: Aβ1-42 levels ↓ (cortex and hippocampus) 5xFAD/Mino: Relative plaque ↓	Morris water maze	5xFAD/FAAH−/−/Mino: escape latency ↑ 5xFAD/Mino: escape latency ↓	5xFAD/Mino: Aβ plaque ↓ 5xFAD/FAAH−/−/Mino: Aβ1-42 levels ↓	-	Aparicio et al. (2018)
			2. WT/FAAH−/−								
			3. 5xFAD								
			4. 5xFAD/FAAH−/−								
			5. 5xFAD/FAAH−/−/Mino								
			(n = 9) each group								
10	Extract of Fructus cannabis ameliorates learning and memory impairment induced by D-galactose in an aging rats model	3-month-old Sprague-Dawley (SD) (male) rats, wt 259–278 g. D-gal at a dose of 400 mg/kg/day via i.p for 14 weeks	1. Control	EFC was administered intragastrically once daily (200 mg/kg/day or 400 mg/kg/day) for 14 weeks (extract with 90% ethanol in 24 hrs)	GFAP expression ↓	SOD ↑; MDA ↓ PS 1 & p-tau ↓	Morris water maze	Escape latency @ D3-D5 ↓; target quadrant time ↑	PS1 ↓, p-tau ↓	-	N. Y. Chen et al. (2017)
			2. EFC (400 mg/kg)								
			3. D-gal								
			4. D-gal + EFC (200 mg/kg)								
			5. D-gal + EFC (400 mg/kg)								
			(n = 8) each group								
11	Activation of CB2 receptor system restores cognitive capacity and hippocampal Sox2 expression in a transgenic mouse model of Alzheimer’s disease	Adult (female) APP/PS1 mice	1. Wild-type	Mice were administered MDA7 14 mg/kg intraperitoneally (i.p.) every other day for 5 months	Iba1 expression ↓ (hippocampal & entorhinal cortex)	Hippocampus: CB2 expression & Aβ plaque ↓; Sox2 ↑ LTP ↑	Morris water maze	Escape latency ↓; target quadrant time ↑	Aβ plaque ↓	-	Wu et al. (2017)
			2. Wild-type + MDA7								
			3. APP/PS1								
			4. APP/PS1 + MDA7 (n = 5) each group								
12	Delineating the efficacy of a cannabis-based medicine at advanced stages of dementia in a murine model	APP/PS1 mice (male) aged 12 months	12 months	Extracts (9-THC 0.75 mg/kg + CBD 0.75 mg/kg) were administered intraperitoneally (i.p.) in a single injection once daily for 5 weeks (supplied by pharmaceutical)	GFAP & Iba1: no significant changes (neocortex)	Somatosensory cortex: PSD- 95 & GABA-A Rα1 ↑; GluR2/3 ↓; SNAP25 ↓; Synaptotagmin, Txn2, & Wnt16: no changes	Two-object recognition test	Recognition index (RI) ↑	Aβ plaque: no difference	-	Aso et al. (2016)
			1. WT + Veh (n = 8)								
			2. WT + -THC + CBD (n = 9)								
			3. AβPP/PS1+Veh (n = 10)								
			4. AβPP/PS1+-THC + CBD (n = 11)								
			3 months								
			1. WT + Veh (n = 7)								
			2. WT + -THC + CBD (n = 8)								
13	Endocannabinoid regulation of amyloid-induced neuroinflammation	6-month-old 5xFAD mice (male/female)	1. WT	Mice received intraperitoneal injections (i.p.) of the FAAH inhibitor (URB) 3 mg/kg (12 consecutive days). Mice with deletion of the gene for FAAH (FAAH−/−) (purchased)	5xFAD/FAAH−/−: GFAP & Iba1 ↓	5xFAD + URB: IL-1β, IL-6 ↓ 5xFAD + SR1: IL1β, IL6, iNOS, TNFα ↑ 5xFAD/FAAH−/−: IL-1β, IL6 ↑; neuritic plaque ↓; APP level ↓ 5xFAD + URB & 5xFAD/FAAH−/−: AEA, PEA, OEA ↑	Morris water maze	Escape latency ↓	Aβ plaque ↓ APP ↓	CB1 receptor antagonist (SR1)	Vázquez et al. (2015)
			2. 5xFAD								
			3. 5xFAD + URB								
			4. 5xFAD + SR1								
			5. 5xFAD + URB + SR1								
			(n = 10) each group								
14	Cannabinoid receptor 2 deficiency results in reduced neuroinflammation in an Alzheimer’s disease mouse model	APP/PS1 mice	9 months and 14 months respectively	APP/PS1 mice were crossed with CB2−/− mice. APP/PS1*CB2þ/mice were then crossed with CB2−/− mice to become APP/PS1*CB2−/− mice	APP/PS1*CB2−/−: Iba1 expression ↓; microglia cell ↓; infiltrating macrophage cell ↓: CD40 ↓	CB2−/−: ICAM-1 ↓; IL-6, TNFa, CCL2 ↓ APP/PS1*CB2−/−: TNFα, CCL2 ↓ 14-month-old APP/PS1*CB2−/−: Ab plaques deposition ↓ 9-month-old APP/PS1*CB2−/−: Aβ40 and Aβ42 level ↓	Morris water maze	6-month-old APP/PS1*CB2−/− & CB2−/−: escape latency ↓	soluble Aβ40 and Aβ42 ↓	-	Schmöle et al. (2015)
			1. Control								
			2. CB2−/−								
			3. APP/PS1								
			4. APP/PS1*CB2−/−								
			(n = 10) each group								
15	Cannabis-based medicine reduces multiple pathological processes in a PP/PS1 mice	APP/PS1 mice (male)	1. WT (n = 7–11)	Extracts of THC, 0.75 mg/kg; CBD, 0.75 mg/kg; THC + CBD, 0.75 mg/kg each was administered intraperitoneally (i.p) as a single injection once daily for 5 weeks (supplied by pharmaceutical)	APP/PS1*THC + CBD/APP/PS1*THC/APP/PS1*CBD: GFAP expression ↓ APP/PS1*THC + CBD: Iba1 expression ↓	APP/PS1*THC + CBD: Aβ42 level ↓ APP/PS1*THC + CBD: Mapk3, Psmb2, Txn2, and Wnt16 genes ↓ APP/PS1*THC + CBD: Txn2 protein ↑ APP/PS1*THC + CBD/APP/PS1*THC: Wnt16 protein ↑	Two-object recognition test; Active avoidance test	APP/PS1*THC: Recognition index (RI) ↑ APP/PS1*Veh & APP/PS1*CBD: active avoidance ↓	Aβ plaque ↓; soluble Aβ42 ↓	-	Aso et al. (2015)
			2. WT*THC								
			3. WT*CBD								
			4. WT*THC + CBD								
			5. APP/PS1 (n = 7–8)								
			6. APP/PS1*THC								
			7. APP/PS1*CBD								
			8. APP/PS1*THC + CBD								
16	β-Caryophyllene ameliorates the Alzheimer-like phenotype in APP/PS1 mice through CB2 receptor activation and the PPARγ pathway	APP/PS1 mice (male)	1. Wt-veh	Animals were orally treated by gavage with 16, 48, or 144 mg/kg of β-caryophyllene (BCP) every morning for 10 weeks starting at the age of 7 months	GFAP & Iba1 expression ↓	Hippocampus & cortex: β-amyloid level ↓ COX-2, TNF-α, IL-1β ↓	Morris water maze	Escape latency ↓; target quadrant time ↑	Aβ plaque ↓	AM630; GW9662	Cheng et al. (2014)
			2. APP/PS1-veh								
			3. APP/PS1-BCP (48 mg/kg)								
			(n = 5–7/group)								
17	Cannabinoid receptor 1 deficiency in a mouse model of Alzheimer’s disease leads to enhanced cognitive impairment	APP23 mice (male)	1. WT (n = 11)	APP23 mice were crossed with CB2+/−mice to obtain APP23/CB1−/− mice	APP23/CB1−/−: GFAP ↓	APP23/CB1−/− APP processing APPfl, CTFa and CTFb ↓ Hippocampus/cortex: amyloid plaque ↓ Synaptophysin and PSD95: No changes	Morris water maze	APP23/CB1−/−: Escape latency ↑; target crossing ↓	Aβ plaque ↓; soluble Aβ40 ↓	-	Stumm et al. (2013)
			2. WT/CB1−/− (n = 9)								
			3. APP23 (n = 12)								
			4. APP23/CB1−/− (n = 7)								
18	Activation of the CB2 receptor system reverses amyloid-induced memory deficiency	Adult (male) Sprague–Dawley rats Aβ1–40 fibrils (10µg/3 L) or 3 µL of artificial cerebrospinal fluid were injected stereotaxically and bilaterally into each hippocampus	1) Control	The Aβ1–40 MDA7 group received bilateral intracerebral microinjection of Aβ1–40 fibrils once and 15 mg/kg MDA7 i.p. daily for 14 days (Aβ1–40 + MDA7)	Aβ1–40 + MDA7 CD11b, GFAP expression ↓	Aβ1–40 + MDA7: IL-1β ↓; CB2R expression ↓; amyloid-β clearance ↓	Morris water maze	Aβ1–40 + MDA7: Escape latency ↓. target quadrant time ↑ Aβ1–40 + MDA7+ AM630: Escape latency ↑. target quadrant time ↓	Aβ1–40 ↓	AM630	Wu et al. (2013)
			2) Aβ1–40								
			3) Aβ1–40 + MDA7								
			4) MDA7								
			5) n = 10/group								
19	CB2 cannabinoid receptor agonist ameliorates Alzheimer-like phenotype in a PP/PS1 Mice	APP/PS1 mice (male)	Pre-symptomatic: (n = 6–10)	Animals treated during the pre-symptomatic and early symptomatic phase received one daily administration for 5 weeks with JWH-133	APP/PS1*JWH-133: Iba1 expression ↓	APP/PS1: CB2 gene expression ↑ APP/PS1*JWH-133: IL-1β, IL-6, TNFα, IFNγ expression ↓ APP/PS1*JWH-133: p38, SAPK/JNK expression ↓; GSK3β ↑; Tau-P ↓; HNE ↓; SOD1	Two-object recognition test; Active avoidance test	APP/PS1*JWH-133: Recognition index pre-symp and symp phase (RI) ↑ APP/PS1*JWH-133: active avoidance pre-symp ↑	Aβ plaque: no difference	-	Aso et al. (2013)
			1. WT-Veh								
			2. WT-JWH								
			3. APP/PS1-Veh								
			4. APP/PS1*JWH-133								
			Early-symptomatic: (n = 6–10)								
			5. WT-Veh								
			6. WT-JWH								
			7. APP/PS1								
			8. APP/PS1*JWH-133								
20	Monoacylglycerol lipase is a therapeutic target for Alzheimer’s disease	5XFAD transgenic (TG) mice (female)	1. WT-Veh	Mice were treated with JZL184 (12 mg/kg) three times per week i.p. starting at 2 months of age for 16 weeks or starting at 4 months of age for 8 weeks	CD11b/OX42 ↓ (cortex) GFAP ↓ (cortex & hippocampus)	Hippocampus & cortex: Aβ, Aβ42 & CTFα/β plaques expression ↓; BACE1 ↓; FJC+ ↓ Hippocampal total dendritic spines (CA 1 & DG) ↑ GluR1, GluR2, NR2A, and NR2B expression ↑	Morris water maze	Average latency ↓; Target quadrant time ↑; crossing target ↑	Aβ plaque ↓ Aβ42 plaque ↓	-	R. Chen et al. (2012)
			2. WT-JZL								
			3. TG-Veh								
			4. TG-JZL184								
			(n = 9–12) per group								
21	WIN55212-2 attenuates amyloid-beta-induced neuroinflammation in rats through activation of cannabinoid receptors and PPAR-g pathway	Wistar rats aged (male) 10–12 weeks. Aβ (1–42) was administered i.h.p. (50 ng)	Sham grp	WIN and other drugs were brought to their final concentration in vehicle prior to use and were administered i.c.v. Treatments started 1 h subsequent to Aβ (1–42) on day 1, 3, 5 and 7	Aβ*WIN: Caspase 3 immunostaining ↓	Aβ*WIN: PPAR-γ ↑; TNFα ↓ NF-κB ↓; caspase 3 ↓	Morris water maze	Target quadrant time ↑	-	GW9662 AM251 SR144528	Fakhfouri et al. (2012)
			1. WT*Veh								
			2. WT*WIN								
			Aβ grp								
			1. Aβ*Veh								
			2. Aβ*WIN								
			3. Aβ*AM + WIN								
			4. Aβ*AM								
			5. Aβ*SR + WIN								
			6. Aβ*SR								
			7. Aβ*GW + WIN								
			8. Aβ*GW								
			9. Aβ*AM + SR + GW + WIN (n = 5) per group								
22	Prolonged oral cannabinoid administration prevents neuroinflammation, lowers β-amyloid levels and improves cognitive performance in Tg APP 2576 mice	TgAPP transgenic mice (male)	1. WT	WIN 55,212-2 (WIN) and JWH-133 (JWH) were administered in the drinking water at a dose of 0.2 mg/kg/day using ethanol (0.1%) as vehicle for 4 months	TgAPP + JWH: Iba1+ ↓ (cortex)	TgAPP + JWH: 18FDG uptake in hippocampus & cortex ↑ TgAPP + JWH: CB2 protein, COX2, TNFα expression ↓; β-amyloid level ↓ TgAPP + WIN: p-Ser9 GSK3b ↑	Novel object recognition test (NOR)	TgAPP + JWH: Recognition index (RI) ↑	Aβ1–40 ↓ Aβ1–42 ↓	-	Martín-moreno et al. (2012)
			2. WT + WIN								
			3. WT + JWH								
			4. TgAPP + Veh								
			5. TgAPP + WIN								
			6. TgAPP + JWH (n = 5–7) per group								
23	CB1 agonist ACEA protects neurons and reduces the cognitive impairment of APP/PS1 mice	Male APP/PS1 mice (male)	1. WT-Veh (n = 6)	Animals treated during the pre-symptomatic phase received (1.5 mg/kg) one daily administration for 5 weeks with ACEA starting at 3 months of age	GFAP (pre-symp & symp) ↓ Iba1 no changes	IFN-γ expression ↓; Tau-p ↓ p-Ser9-GSK3b ↑	Two-object recognition test; Active avoidance test	Recognition index pre-symp and symp phase (RI) ↑	Aβ plaque ↓ (cortex)	Rimonabant	Aso et al. (2012)
			2. WT-ACEA (n = 10)								
			3. AβPP/PS1-Veh (n = 7)								
			4. AβPP/PS1-ACEA (n = 9)								
			(n = 3–6) per group								
24	Cannabidiol and Other Cannabinoids reduce microglial activation in vitro and in vivo: relevance to Alzheimer’s disease	C57/Bl6 mice (male) of 3 months of age were intraventricularly injected with 2.5 µg of fibrillar Aβ or saline 5 µg	1. SCR + Veh	Mice were administered with Intraperitoneal treatment with the cannabinoids (20 mg/kg CBD; 0.5 mg/kg HU-308, JWH, and WIN). During the first week, the mice were treated daily, then for 2 weeks, they were treated 3 days/week	Microglia intracellular Ca (CBD, WIN, JWH) ↓ Microglia migration (CBD, WIN, JWH, HU) ↑	IL-6 expression (WIN, CBD) ↓	Morris water maze	Escape latency ↓	-	SR1; SR2	Martín-Moreno et al. (2011)
			2. Fib + Veh								
			3. Fib + WIN								
			4. Fib + CBD								
			(n = 8) per group								
25	Cannabinoid receptor stimulation is anti-inflammatory and improves memory in old rats	Eighteen young (3 months old) and 24 old (23 months old) (male F-344 rats) (Harlan Sprague–Dawley, Indianapolis) – aging-related model	1. Young + vehicle (n = 6)	WIN-55212-2 were chronically infused for 21 days subcutaneously using an osmotic minipump	Old + WIN 2 mg/kg day: activated microglia ↓	Strong spatial co-localization of CB1 receptors and NMDA-R1 receptors on neuronal cell bodies and dendritic processes Hippocampal CB1 receptor binding assay: young > old	Water pool testing (Morris water maze)	Latency ↓	-	-	Marchalant et al. (2008)
			2. Old + vehicle (n = 8)								
			3. Young + WIN 0.5 mg/kg day (n = 6)								
			4. Old + WIN 0.5 mg/kg day (n = 8)								
			5. Young + WIN 2 mg/kg day (n = 6)								
			6. Old + WIN 2 mg/kg day (n = 8)								
26	Prevention of Alzheimer’s disease pathology by cannabinoids: neuroprotection mediated by blockade of microglial activation	βA25–35 was injected to Wistar rats (male) intracerebroventricularly daily for 7 days	1. SCR + Veh	Animals received a cannabinoid (WIN55,212-2, 10 µg in 10 µL of 20% DMSO/80% saline per day (7 days)	βA25–35 + WIN: microglial cell ↓	βA25–35 + WIN: CB1 expression ↓; calbidin ↓	Morris water maze	βA25–35 + WIN: Escape latency ↓	-	-	Ramírez et al. (2005)
			2. SCR + WIN								
			3. βA + Veh								
			4. βA + WIN (n = 5) per group								

#### 3.2.1 Animal models

The selected studies used two kinds of animals: rats (8 studies) and mice (18 studies; 16 transgenic and two wild-type or non-transgenic). The number of animals used in each group of the included studies ranged between 5 and 15 animals per group. However, no study described the method for determining the sample size. Eighteen studies used male animals, four used female animals, and two used both male and female animals. The 26 included studies involved six types of induction leading to AD rodent models, including i) stereotaxic injection of Aβ or streptozotocin (STZ); ii) transgenic mice such as familial AD (FAD)/TgAPP/APP/PS1/tau-transgenic; iii) transgenic crossed with CB2^−/−^ mice, iv) aging-related animal models; v) D-galactose alone or combined with aluminum chloride (AlCl_3_)-induced AD; and vi) D-galactose combined with ovariectomy.

Amyloid peptide AD induction *via* direct intracerebral injection into distinct brain regions causes deficits in learning and memory abilities, which trigger behavioral changes comparable to those seen in AD. However, this treatment does not mimic the slow neurodegenerative process because it is an acute toxicity model. The intracerebroventricular (i.c.v) microinjection of Aβ (1–42) into the hippocampus was performed as a single injection on the first day (Martín-Moreno et al., 2011), daily for 7 days (Ramírez et al., 2005), repeated every other day on days 3, 5, 7 (Fakhfouri et al., 2012; Patricio-Martínez et al., 2019), and daily for 14 days (Wu et al., 2013). Meanwhile, streptozotocin (STZ) is another neurotoxin agent administered through i.c.v injections to establish the AD model (Xiang et al., 2022). Following the tau or Aβ-dependent increase in CB2R expression, four studies used genetic mouse models to determine if deletion of CB2 encoding cnr2 genes also modulated disease vulnerability (Schmöle et al., 2015; 2018) and provided neuroprotection (Galán-Ganga et al., 2021; J; Zhang & Chen, 2018). Conversely, APP23 mice with CB1 deficiency showed cognitive deficits despite a reduction in APP processing accompanied by lowered plaque load (Stumm et al., 2013). D-galactose, a neurotoxic agent that induced AD (Chen et al., 2017) resulted in cognitive impairments and neurodegeneration in rats, but lacked AD-related pathological hallmarks. The combination of D-galactose + AlCl_3_ resulted in obvious alterations in the CA1 sub-field of the model group, with disruption of the pyramidal cell layer and cellular degeneration (Mahdi et al., 2021). Evaluation of the restoration of cognitive impairment by CB2R activation was reported in bilaterally ovariectomized female rats administered D-galactose injections (Abd El-Rahman & Fayed, 2022). The anti-inflammatory and memory-enhancing effects of CB1/2 receptor stimulation in normal-aged rats were also investigated since natural aging is associated with increased microglial activation (Marchalant et al., 2008).

Transgenic (Tg) mouse-based models have become mainstream in pre-clinical research on AD. FAD, TgAPP, APP, PS1, and tau-transgenic models are best suited for quantifying amyloid plaque burden and were used in most of the selected studies. Among 16 transgenic mice studies, APP/PS1 was the most used animal model (9 studies), followed by 5xFAD (4 studies). APP23, TgAPP, and hTau models were used in one study each. APP-based animals were the first approach to reproduce and analyze the histopathological progression of cerebral Aβ (Sanchez-Varo et al., 2022). APP/PS1 was reported in Swedish mutant mice with L166P mutation in the presenilin 1 gene (Li et al., 2019), whereas a new generation of double Tg mice (5xFAD) co-expressed human APP and PS1 or PS2 mutations (Aparicio et al., 2018). A TgAPP mice model expressed the human APP containing a double mutation (Lys 670-Asn/Met 671-Leu) (Martín-Moreno et al., 2012), while an APP23 mice model showed hemizygous expression of the AD-linked KM670/671NL double mutation (Swedish mutation) of human APP (Stumm et al., 2013). Only one tauopathy model was included, in which the six isoforms of wild-type (WT) human tau were expressed in a mouse model with hTau (Galán-Ganga et al., 2021).

#### 3.2.2 Intervention characteristics

The cannabinoids agents used in the studies included WIN55,212-2, a non-selective CBR agonist (Marchalant et al., 2008; Fakhfouri et al., 2012; Mahdi et al., 2021); selective CB2R agonists such as AM1241 (Abd El-Rahman & Fayed, 2022); JWH-015 (Li et al., 2019), MDA7 (Wu et al., 2013; 2017); β-caryophyllene (Cheng et al., 2014); JWH-133 (Aso et al., 2013); ACEA a CB1R agonist (Patricio-Martínez et al., 2019); MAGL inhibitors like JZL184 (R. Chen et al., 2012); and FAAH inhibitors like URB (Vázquez et al., 2015). Other agents included tetrahydrocannabinol (THC) as a psychoactive, cannabidiol (CBD) as a non-psychoactive, or a combination of THC and CBD (Aso et al., 2016). Some studies used atypical CBRs like O-1602 (Xiang et al., 2022) or *C. sativa* leaf extract (Chen et al., 2017).

#### 3.2.3 Effects of endocannabinoid-mediated glial cells in AD models

(1) Cognitive assessment

All the included studies performed behavioral tests to assess animal learning and memory function. These tests included a two-object recognition test alone and with an active avoidance test (4 of 26), a novel object recognition test (NOR) (5 of 26), Morris water maze (MWM) tests (16 of 26), and eight-arm radial tests (1 of 26). We separated the data into three categories according to different animal model species such as “wild-type” (Martín-Moreno et al., 2011; Xiang et al., 2022), “transgenic mice” (Aparicio et al., 2018; Aso et al., 2012; Aso et al., 2013; Aso et al., 2015; Aso et al., 2016; R; Chen et al., 2012; Cheng et al., 2014; Galán-Ganga et al., 2021; C; Li et al., 2019; Martín-moreno et al., 2012; Schmöle et al., 2015; Schmöle et al., 2018; Stumm et al., 2013; Vázquez et al., 2015; Wu et al., 2017; J; Zhang and Chen, 2018) and “rats” (Abd El-Rahman and Fayed, 2022; N. Y; Chen et al., 2017; Fakhfouri et al., 2012; Mahdi et al., 2021; Marchalant et al., 2008; Patricio-Martínez et al., 2019; Ramírez et al., 2005; Wu et al., 2013) to reduce variability across groups for analysis. We used ImageJ (Rawak Software Inc., Stuttgart, Germany) and RevMan 5.4 to quantify and analyze the data from four included studies. To investigate the protective impact of endocannabinoid-mediated glial cells on escape latency in MVM, novel object recognition, pathological determination, glial cell activation, and anti-neuroinflammation action, we performed a meta-analysis of the outcome and displayed the data *via* a forest plot.

**FIGURE 3:**
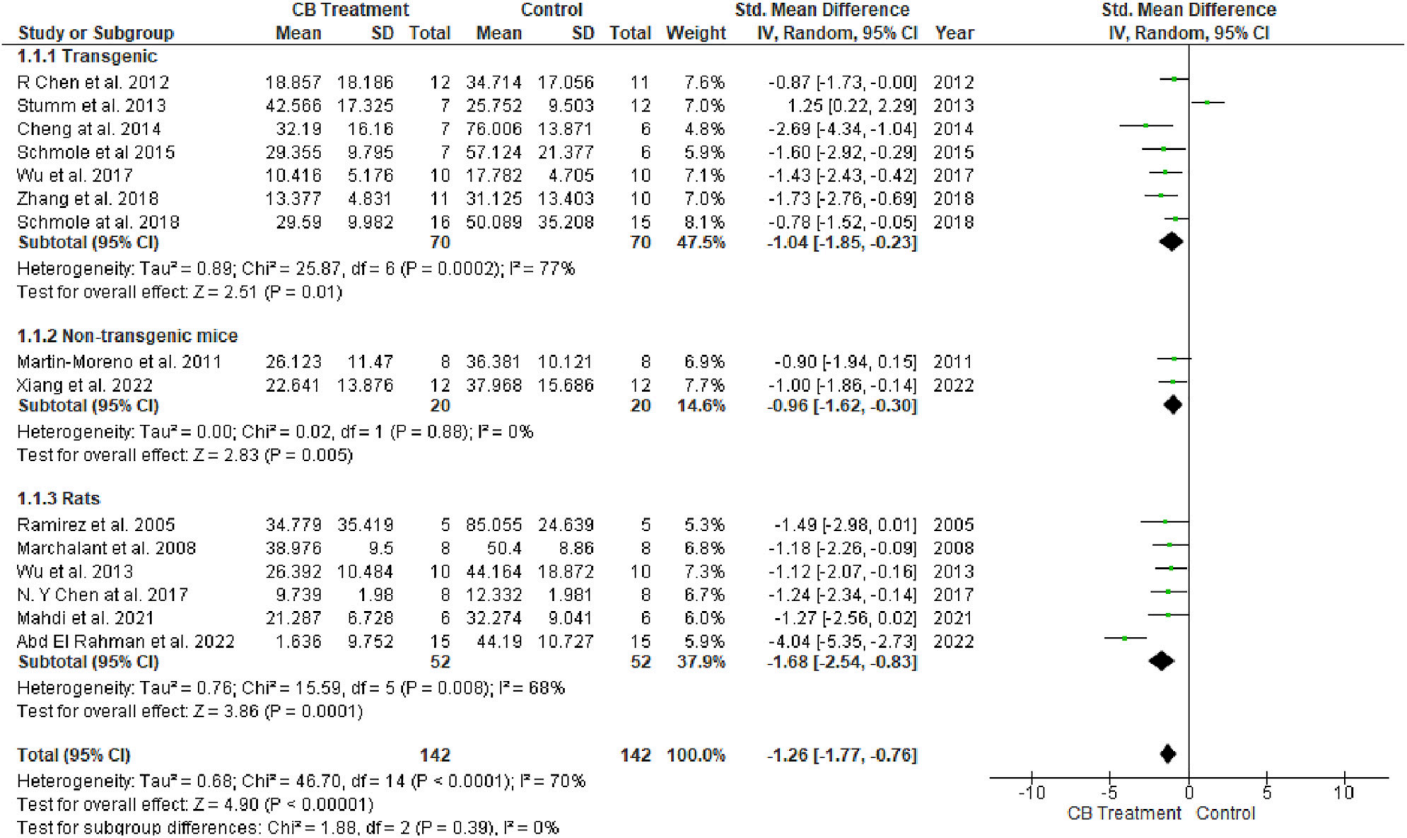
Forest plot for comparison: Cannabinoid *versus* AD model. Outcome: escape latency.

The MWM test is a popular method used to assess spatial learning and memory. It is utilized in AD models to test cognitive function, memory, and medication efficacy. After training days, the MWM probe test is performed by removing the platform and allowing the tested animals to swim freely for 60 s. The amount of time spent in the target quadrant (where the platform is located on the training days) and the number of times the animal passes through it are assumed to signify the degree of memory consolidation during training. Six of 16 studies used rats as models, while the remaining 10 studies used mice. A total of 142 animals were treated with various cannabinoids at different doses and durations ranging from acute administration up to 5 months of chronic treatment (Wu et al., 2017). Another 142 vehicle-treated animals were added as controls. Univariate statistical analysis in all three subgroups (Figure 3) using a random-effects model showed that cannabinoid treatment significantly reduced the time to escape from the platform according to the overall effect size (SMD = −1.26; 95% CI: −1.77 to −0.76, *p* < 0.00001). The highest heterogeneity belonged to the transgenic mice group (77%, *p* = 0.0002). No heterogeneity was observed in the non-transgenic mice (wild-type) subgroup (I^2^ = 0%, *p* = 0.88), while moderate heterogeneity was seen in the rat subgroup (I^2^ = 68%, *p* = 0.008).

**FIGURE 4:**
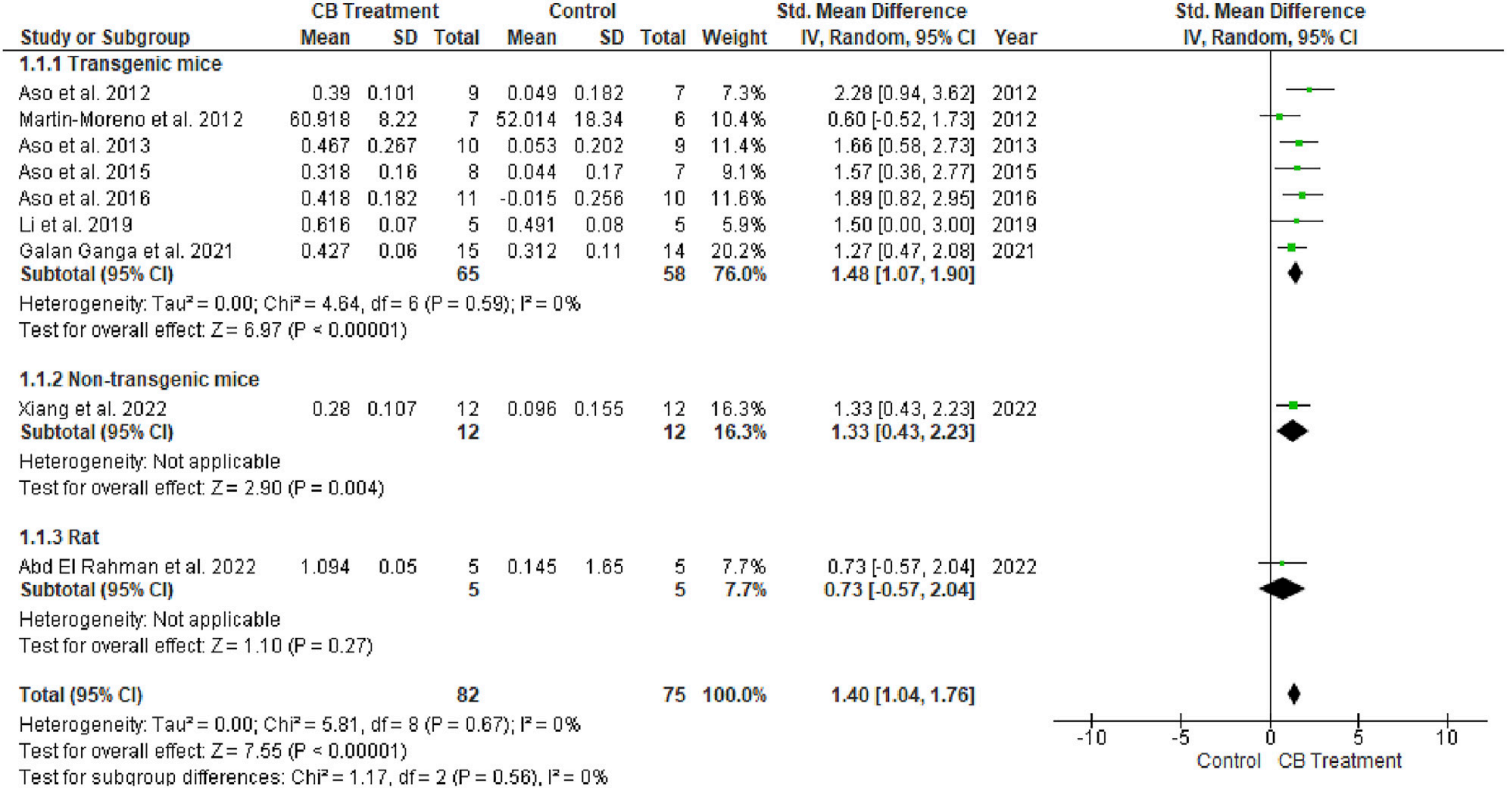
Forest plot for comparison: Cannabinoid *versus* AD model. Outcome: recognition index.

The novel object recognition (NOR) test is used to assess recognition memory. An investigator blinded to the treatment groups conducts the exam over 3 days. On day one, the mice are acclimatized for 20 min to the empty arena. On day 2, mice are returned to the arena for 6 minutes with two identical objects. On the third day, one of the two familiar objects is replaced with a new object of a different material, color, and shape. Six minutes are given for mice to explore the arena and the total time spent exploring each object is recorded. Exploration is defined as nodding and sniffing at an object from a distance of <2 cm. The details are presented in the form of a recognition index (RI). Rodents administered cannabinoid agonists better discriminated the novel object compared to the vehicle-treated group according to the overall effect size (SMD = 1.40; 95% CI: 1.04 to 1.76, *p* < 0.00001). Interestingly, no heterogeneity was observed (I^2^ = 0, *p* = 0.76) (Figure 4).

(2) Pathological determination

**FIGURE 5:**
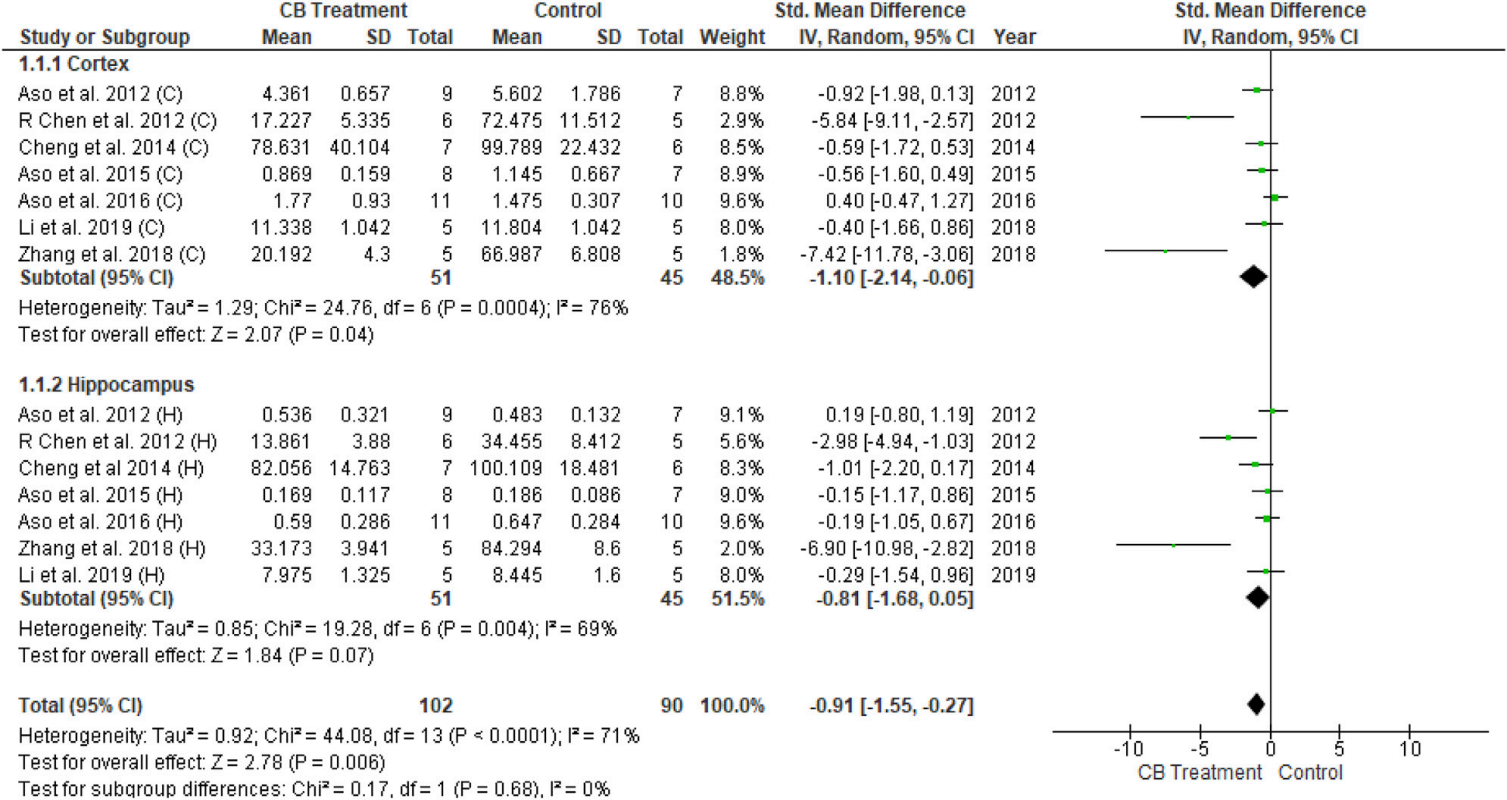
Forest plot for comparison: Cannabinoid *versus* AD model. Outcome: amyloid plaques.

The amyloid hypothesis proposes that regardless of hereditary or sporadic condition, senile plaques develop in the brain due to amyloid peptide accumulation and aggregation. Fourteen of the 26 studies described the pathological assessments of Aβ burden in mice, in which all Aβ plaques formed in transgenic mice. Two studies involved FAAH inactivation (FAAH−/−) while another two studies featured cannabinoid receptor deficiency (CB1−/− and CB2−/−, respectively). The remaining studies used transgenic mice to produce Aβ plaques. The amount of plaques was determined from immunohistochemical analysis and was analyzed using RevMan 5.4. With moderate heterogeneity (I^2^ = 71 percent, *p* < 0.0001), univariate statistical analysis revealed a significant decrease in Aβ plaques according to the overall effect size (SMD = −0.91; 95% CI: −1.55 to −0.27, *p* = 0.006) toward the endocannabinoid-treated group over the vehicle-control group (Figure 5). The results of subgroup analysis indicated that endocannabinoid treatment significantly decreased Aβ plaques in the cortex (*p* < 0.04) compared to the hippocampus (*p* = 0.07).

The two most common Aβ species end at residues 40 and 42; the latter exhibits a stronger propensity for aggregation and is markedly more neurotoxic due to the presence of two additional hydrophobic amino acids. Three of the 26 studies reported Aβ oligomeric assessments such as Aβ1–42 pathology; another three studies reported on soluble Aβ40 and Aβ42.

#### 3.2.4. Mechanisms of endocannabinoid-mediated glial cells in AD models

(1) Glial cell activation

Twenty of the 26 selected studies ran immunoreaction tests with either Iba1 as a marker for microglia, GFAP as a marker for astrocytes, or both immunoreaction markers. Of these 20 studies, six conducted assessments of both GFAP and Iba1, eight assessed GFAP only, and six assessed Iba1 only. The remaining six studies conducted other microglial investigations such as microglial phenotyping, cell numbers, and macrophage infiltration instead of markers of glial cell immunoreaction. Besides the Iba1 marker, other microglial markers (CD68 and CD11b) were also applied. Furthermore, the CD68 and CD11b microglial markers were co-assessed with GFAP and they simultaneously reduced with cannabinoid treatment. One study reported that CD68 microglia expression was reduced following AM1241 administration (Abd El-Rahman and Fayed, 2022). Genetically CB2-deleted transgenic mice showed decreased microglial CD40 expression compared with normal transgenic (Schmöle et al., 2015). Decreased CD11b microglial marker was observed following MDA7 treatment in Aβ1–40 fibrils injected rats (Wu et al., 2013) and JZL184 treatment in 5XFAD transgenic mice (R. Chen et al., 2012). There was one study that indirectly used active caspase 3 to represent the presence of glial cells marked by positive-immunoreactivity paralleled with enhanced active caspase 3 immunostaining (Fakhfouri et al., 2012).

**FIGURE 6:**
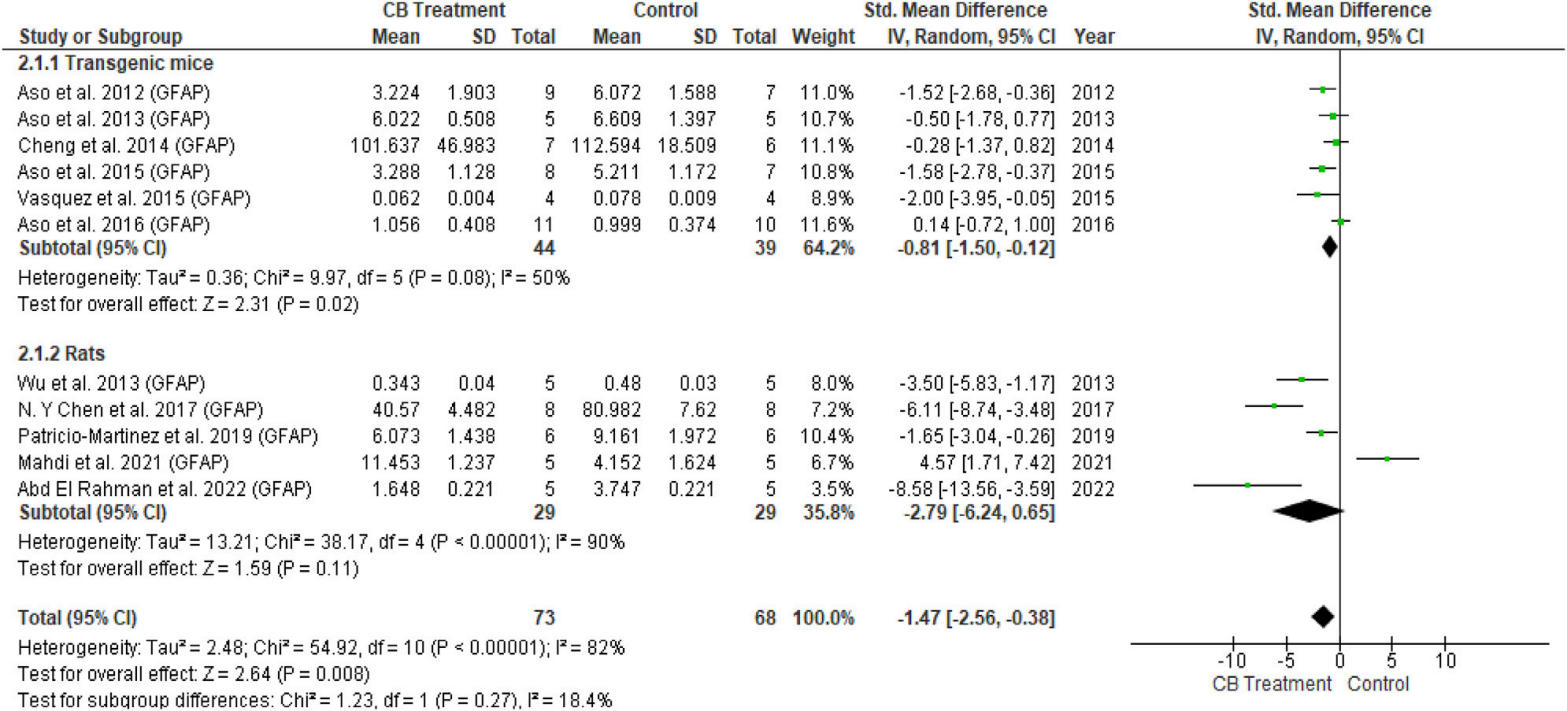
Forest plot for comparison: Cannabinoid *versus* AD model. Outcome: GFAP.

**FIGURE 7:**
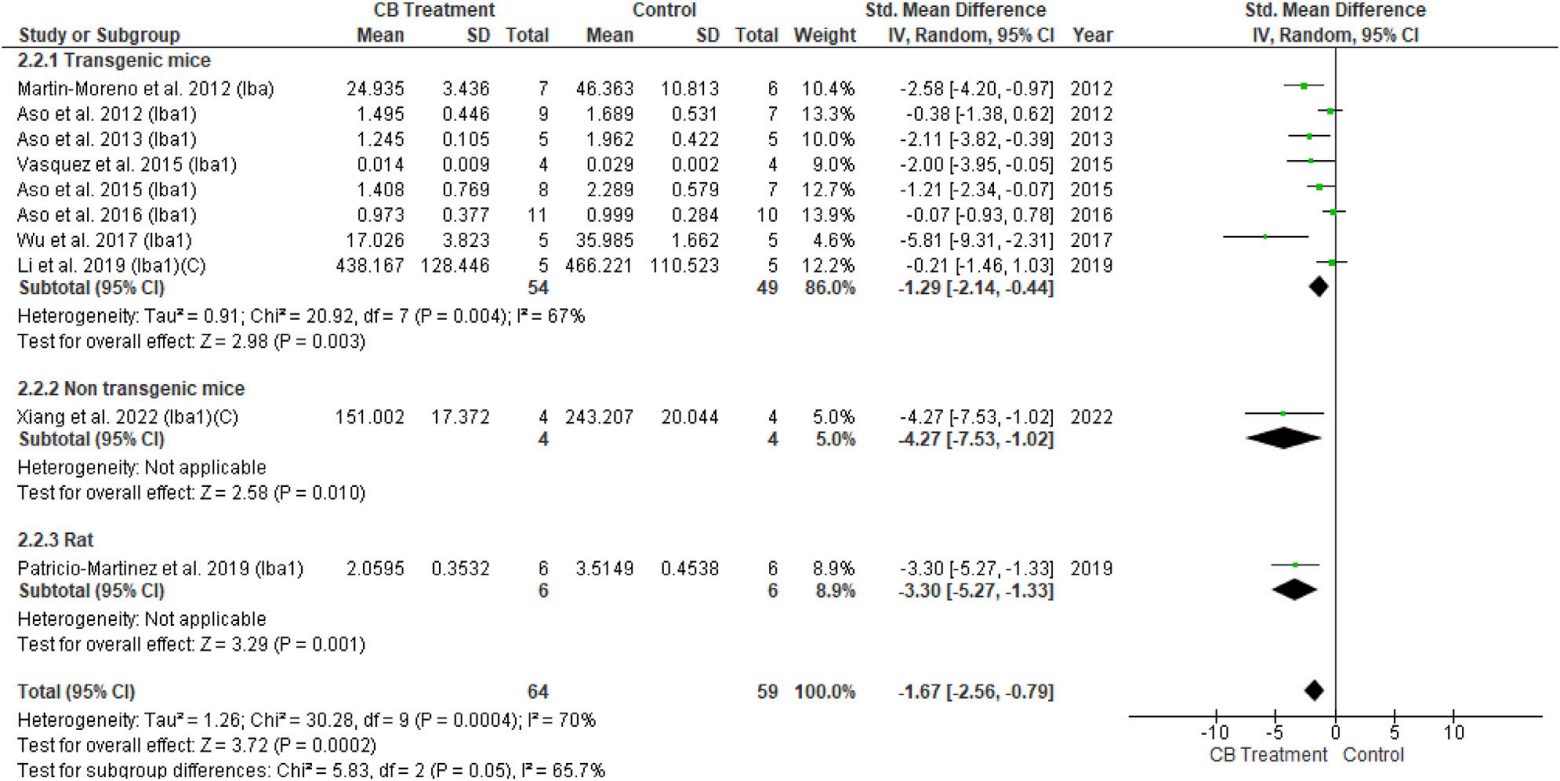
Forest plot for comparison: Cannabinoid *versus* AD model. Outcome: Iba1.

Iba and GFAP expression levels were increased in the AD model compared with normal vehicle administration animals. With cannabinoid stimulation, our analysis found that nine of 11 overall studies involving Iba1 reported its reduced expression compared to the control, while two studies showed non-significant changes. Similarly, following cannabinoid treatment, 11 of 14 studies reported reduced GFAP expression compared to the control; two other studies showed a non-significant result, and one reported increased GFAP expression. The non-significant difference in glial cell activity between the cannabinoid and control groups appeared when AβPP/PS1 mice were treated at advanced stages (Aso et al., 2016). All six studies combining Iba1 and GFAP observed decreased levels for both markers following cannabinoid administration. The results of univariate statistical analysis revealed a significant decrease in GFAP in the endocannabinoid-treated group compared to the vehicle-controlled group with an overall estimate (SMD = −1.47; 95% CI: −2.56 to −0.38, *p* = 0.008) −1.28 and substantial heterogeneity (I^2^ = 82%, *p* = 0.00001) (Figure 6). Similarly, Iba1 expression was significantly decreased in the endocannabinoid-treated group compared to the vehicle-controlled group according to the overall effect (SMD = −1.67; 95% CI: −2.56 to −0.79, *p* = 0.0002) with moderate heterogeneity (I^2^ = 70%, *p* = 0.0004) (Figure 7). Finally, the remaining six of 25 studies that performed microglia investigations reported decreased microglial number (Ramírez et al., 2005) (Aparicio et al., 2018), activity (Fakhfouri et al., 2012) and infiltration (Schmöle et al., 2015), as well as a stable morphology (Schmöle et al., 2018; Galán-Ganga et al., 2021). Although not included in the statistical analysis in this review due to their distinctive evaluation of glial cells, the decrease in microglial activity was observed as a response to endocannabinoid modulation.

(2) Anti-neuroinflammatory action

Fourteen of 26 studies were conducted on neuro-inflammatory markers, including eight studies using transgenic mice and six studies using wild-type mice or rats. A total of 76 animals received different cannabinoid agents at different doses, while 74 vehicle-treated animals were added as controls. Two studies each used CB2R and FAAH knockout mice, respectively. Most studies assessing inflammatory parameters showed a significant reduction in pro-inflammatory cytokine levels following cannabinoid administration, indicating the anti-inflammatory effect of cannabinoids by repressing activated microgliosis and astrocytosis. The evaluated variables included IL1β (7 studies), IL6 (7 studies), tumor necrosis factor-alpha (TNF-α) (10 studies), IFN-γ (2 studies), NFκB p65 (3 studies), and COX2 (2 studies). Out of 14 studies that assessed inflammatory cytokines, 11 reported on more than one cytokine, with the remaining three studies reporting only one cytokine. Of the 14 studies reporting neuroinflammation measurement, 11 were involved with cannabinoid agonist administration, one study concerned CB2R deletion while the remaining two studies utilized FAAH deletion animal models. Microglia and astrocytes are essential modulators of neuroinflammation in the central nervous system, responding quickly to infections, stress, and injury. Microglia and astrocyte activation leads to neuroinflammation-mediated neurodegeneration in the pathological development of AD, which will be elaborated on later.

Among 14 studies reporting neuroinflammatory markers in the endocannabinoid modulation of glial cells, 11 described a reduction in neuroinflammation in the treatment group compared to the control group. However, three other studies reported the opposite in transgenic (male) mice overexpressing hTAUP301S with concurrent CB2R deletion (Galán-Ganga et al., 2021), and FAAH knockout mice (Vázquez et al., 2015; Aparicio et al., 2018). These controversial findings are, at present, hard to translate, but could be due to animals with CB2R or FAAH deletions. Therefore, sensitivity analysis was performed in the present study by excluding the most outliers to control for heterogeneity and overall effect. The inflammatory mediators extracted for data analysis were TNF-α, IL-1β, and IL-6. Overall, 21 measurements of cytokines were reported, among which only five described increased neuroinflammation.

**FIGURE 8:**
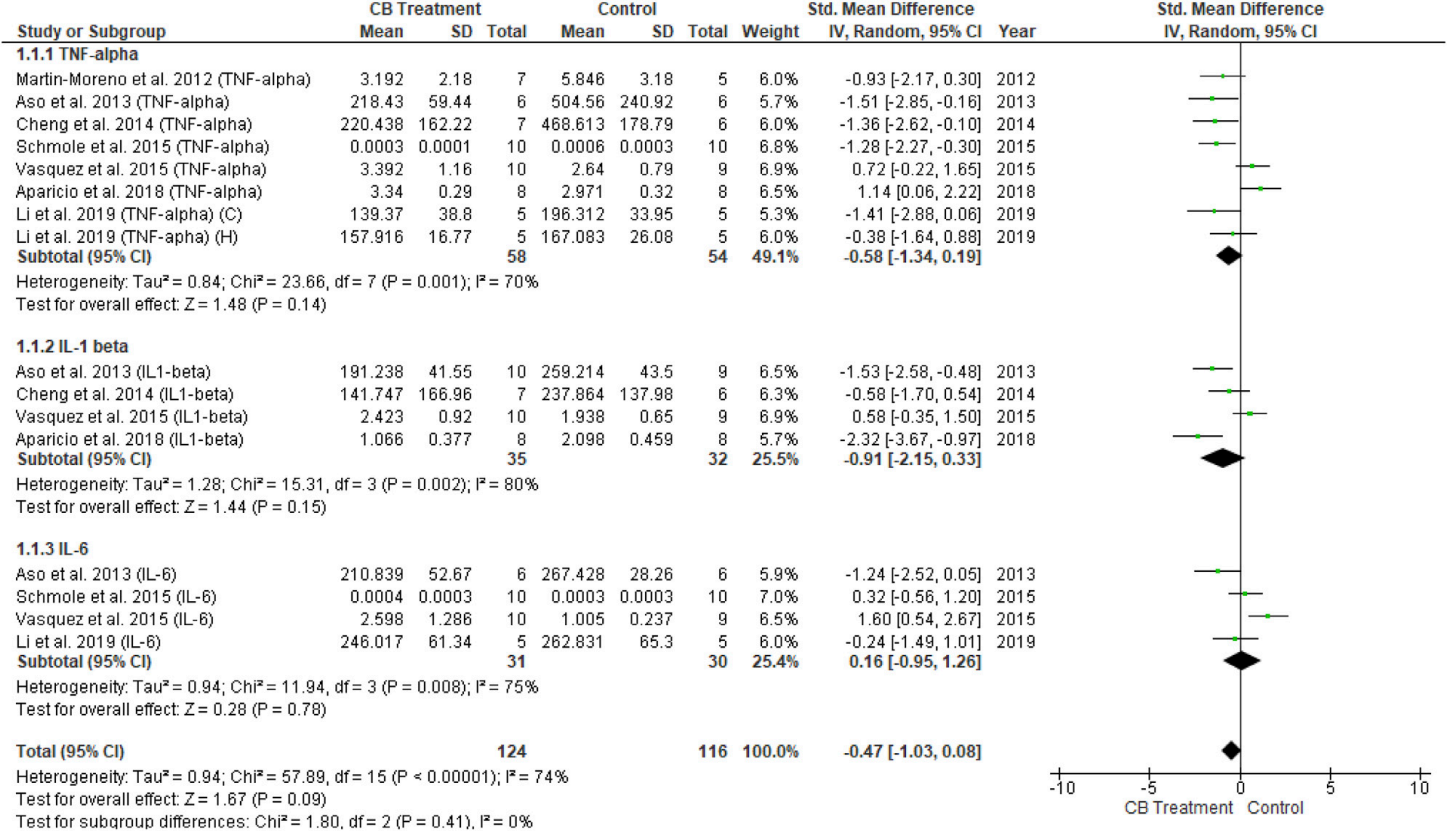
Forest plot for comparison: Cannabinoid *versus* AD model. Outcome: pro-inflammatory cytokines in transgenic mice.

**FIGURE 9:**
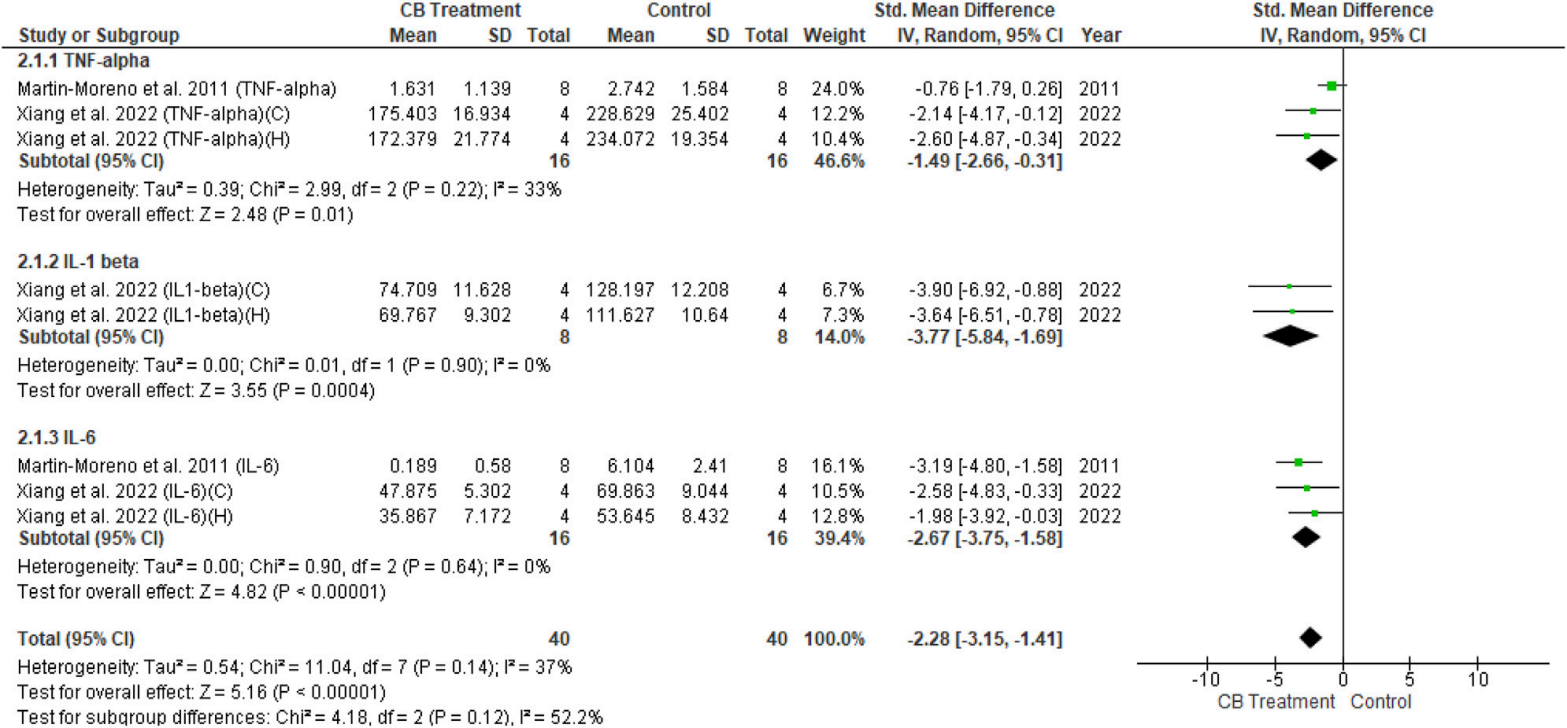
Forest plot for comparison: Cannabinoid *versus* AD model. Outcome: pro-inflammatory cytokines in wild type mice.

The univariate analysis of transgenic mice included six studies. The results revealed a non-significant decrease in all three cytokines (*p* = 0.09) in the endocannabinoid-treated group compared to the vehicle-controlled group according to the overall effect (SMD = −0.47; 95% CI: −1.03 to 0.08, *p* = 0.09) with nearly-substantial heterogeneity (I^2^ = 74%, *p* = 0.00001) (Figure 8). Three studies of wild-type mice included in univariate statistical analysis showed significantly reduced levels of all three cytokine types (SMD = −2.28; 95% CI: −3.15 to −1.41, *p* = 0.00001) in the endocannabinoid-treated group compared to the vehicle-controlled group with moderate heterogeneity (I^2^ = 37%, *p* = 0.14) (Figure 9).

(3) Other endocannabinoid mechanisms of action

Endocannabinoid-mediated glial cells orchestrate alterations in biochemical expression toward neuroprotection, representing cognitive amelioration. Parameters relevant to neuroinflammatory and other mechanisms of AD involving several tests were recorded. Evaluations of oxidative stress, including those for nestin (Mahdi et al., 2021), NO • and nitric oxide synthase (iNOS) (Li et al., 2019; Patricio-Martínez et al., 2019; Vázquez et al., 2015), superoxide dismutase (SOD) (Aso et al., 2013; N. Y; Chen et al., 2017; Mahdi et al., 2021), and malondialdehyde (MDA) (N. Y. Chen et al., 2017; Xiang et al., 2022) expression were observed as these play pivotal synergistic roles in the pathogenesis of neurodegenerative processes, particularly in AD. Other mechanisms involve the expression of cAMP response element-binding protein (CREB) (Abd El-Rahman and Fayed, 2022), brain-derived neurotrophic factor (BDNF) (Galán-Ganga et al., 2021), PSD-95 (Aso et al., 2016; J; Zhang and Chen, 2018) (Stumm et al., 2013), and long-term potentiation (LTP) (R. Chen et al., 2012; Wu et al., 2013; Wu et al., 2017) for the assessment of neurogenesis and synaptic plasticity.

Caspase 3 (Fakhfouri et al., 2012) and Fluoro-jade C (J. Zhang and Chen, 2018) (R. Chen et al., 2012) as markers of apoptosis and degeneration of neurons were also evaluated. The expression of sex-determining region Y-box 2 (Sox2) (Wu et al., 2017), mitogen-activated protein kinase 3 (MAPK3), proteasome subunit, beta type, 2 (PSMB2), thioredoxin 2 (Txn2), and wingless-related integration site 16 (Wnt16) genes (Aso et al., 2015) were maintained following the administration of cannabinoid agents. The expression of membrane-tethered disintegrin and metalloproteases (ADAM17) responsible for the release of soluble TNF-α (Schmöle et al., 2018) and p-Ser9-GSK3b, which mediates tau’s hyper-phosphorylation (Aso et al., 2012; Martín-Moreno et al., 2012) were also investigated. However, those parameters were not used for univariate statistical analysis since each of them was not a component in the outcome criteria of this review, nor did all studies provide the same parameters.

### 3.3 Methodological quality assessment

Based on the SYRCLE risk of bias tool to evaluate the methodological quality of the 26 animal studies, the number of items with low risk of bias divided by the total number of items was used to determine each study’s quality score (Table 3). The quality scores were in the range of 0%–60%. Only one of the 26 included studies showed low bias risks in six domains (60%), while three studies scored 50%. According to the bias type, only the baseline characteristic domain achieved a score >50%. The scores were rated based on ‘yes’ answers.

**TABLE 3:** Methodological quality of the included studies. A, sequence generation; B, baseline characteristics; C, allocation concealment; D, random housing; E, blinded intervention; F, random outcome assessment; G, blinded outcome assessment; H, incomplete outcome data; I, selective outcome reporting; J, other sources of bias. Y: yes; N: no; NC: not clear.

Study	A	B	C	D	E	F	G	H	I	J
Abd El Rahman and Fayed. (2022)	NC	Y	NC	NC	NC	Y	Y	NC	Y	NC
Xiang et al. (2022)	Y	Y	NC	NC	NC	NC	NC	NC	NC	Y
Galán-Ganga et al. (2021)	Y	NC	NC	NC	Y	Y	Y	Y	NC	Y
Mahdi O et al. (2021)	Y	Y	NC	NC	NC	NC	NC	NC	Y	NC
Patricio-Martínez et al. (2019)	Y	Y	NC	NC	NC	N	NC	N	NC	NC
Li C et al. (2019)	Y	NC	NC	NC	Y	Y	Y	NC	Y	NC
Zhang and Chen (2018)	NC	NC	Y	Y	NC	NC	NC	NC	NC	NC
Aparicio et al. (2018)	NC	NC	NC	NC	NC	NC	NC	NC	Y	NC
Schmöle et al. (2018)	NC	Y	NC	NC	NC	NC	NC	NC	NC	N
N. Y Chen et al. (2017)	NC	Y	Y	NC	NC	NC	NC	NC	NC	NC
Wu et al. (2017)	Y	Y	NC	NC	Y	NC	Y	NC	NC	NC
Aso et al. (2016)	Y	Y	NC	NC	NC	Y	Y	NC	NC	NC
Vázquez et al. (2015)	NC	N	NC	NC	NC	NC	NC	NC	NC	NC
Schmöle et al. (2015)	NC	NC	NC	NC	NC	NC	NC	NC	NC	NC
Aso et al. (2015)	Y	Y	NC	NC	Y	Y	Y	NC	NC	NC
Cheng et al. (2014)	NC	Y	NC	NC	NC	NC	NC	Y	NC	NC
Stumm et al. (2013)	NC	NC	NC	NC	NC	Y	Y	NC	NC	NC
Wu et al. (2013)	NC	NC	NC	NC	Y	NC	NC	NC	NC	NC
Aso et al. (2013)	Y	Y	NC	NC	Y	Y	Y	NC	NC	NC
R. Chen et al. (2012)	NC	NC	NC	NC	NC	NC	NC	NC	NC	NC
Fakhfouri et al. (2012)	NC	Y	NC	NC	NC	Y	Y	NC	NC	NC
Martín-Moreno et al. (2012)	NC	NC	NC	NC	NC	NC	NC	NC	NC	NC
Aso et al. (2012)	NC	Y	NC	NC	NC	NC	NC	NC	NC	NC
Martín-Moreno et al. (2011)	NC	NC	NC	NC	NC	N	N	NC	NC	N
Marchalant et al. (2008)	NC	Y	NC	NC	N	NC	NC	NC	NC	NC
Ramírez et al. (2005)	NC	NC	NC	NC	NC	N	N	NC	NC	N

**FIGURE 10:**
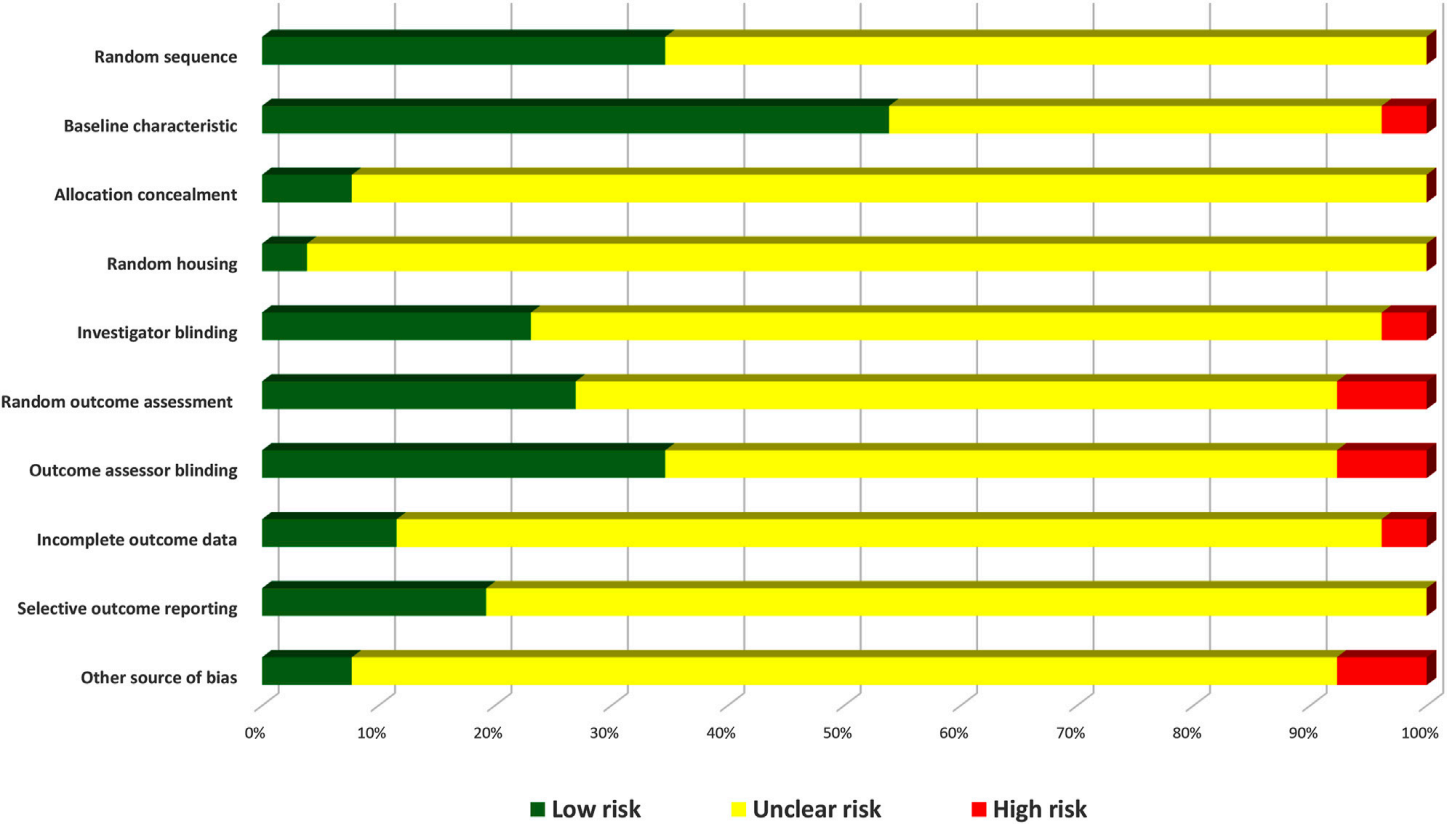
Risk of bias graph of the included pre-clinical studies.

The low-risk scores were generally low in most studies, which could be attributed to the large number of uncertain scores due to a lack of information as most of the studies did not describe in detail each component of bias in their methodology, even though the authors might have performed some of the bias surveillance checklists on the animals during their laboratory work. This is the reason why the uncertain answer covers most of the domains. Overall, the high risk of bias showed the lowest score among the three bias indicators in all domains (Figure 10).

## 4 Discussion

This literature review presents the first systematic analysis of pre-clinical data on the modulation of glial cells by cannabinoids that affects cognitive performance. Evidence of the impact of glial cell endocannabinoid signaling on cognitive deficits in AD was demonstrated by the attenuation of memory impairment (Dominicis et al., 2022). The summary of the glial cell endocannabinoid mechanisms in cognition extracted from each article is presented in Supplementary Table S2.

### 4.1 Potential mechanisms underlying glial cell-endocannabinoid interactions

Studies applying AD animal models have demonstrated the great potential of the endocannabinoid system to control neurodegeneration. Interestingly, the endocannabinoid system’s interaction with glial cells fundamentally determines its role. The neurochemical and biomolecular reaction of glial cells modulated by the endocannabinoid system maintained the stability of the brain/neural microenvironment and ensured neural cell resilience and ultrastructure. This neuro-immunomodulation of glial cells by endocannabinoids eventually displayed the behavioral and cognitive responses that can then be observed and translated into the improvement of the symptoms of dementia, which impair learning and memory in AD.

Clinical practice typically focuses on symptomatic treatment by prescribing cholinesterase inhibitors like donepezil and partial NMDA receptor antagonist-like memantine. Most of these AD treatments target a single pathogenic route; however, some treatments have undesirable side effects (Alzheimer’s Association Report, 2020). In contrast, natural products are characterized by their ability to effectively target numerous pathological disease pathways while having fewer negative effects (Paes-Colli et al., 2022). Overall, the administration of cannabis plants containing cannabinoid agonists improves cognitive deficits in several domains (Amanullah et al., 2017). Although cognitive neurobiology is not fully understood, a large body of data suggests the involvement of several brain networks, with intricate interactions between distinct signaling systems (Rapaka et al., 2021). Therefore, it is unlikely that the endocannabinoid-mediated glial mechanism of action can be wholly attributed to a single specific signaling pathway. Indeed, the pre-clinical studies discussed in this review demonstrate that exogenous cannabinoid treatment for cognitive deficits affects biochemical parameters such as inflammatory and oxidative stress markers, receptor subunit proteins, and signaling proteins. The roles of these systems in the potential mechanisms underlying the effect of cannabinoids on cognitive function are discussed below.

### 4.2 Glial cell-endocannabinoid influences on cognitive changes

In animal studies, the restoration of cognitive/behavioral function from endocannabinoid modulation involves several strategies ranging from observation of endocannabinoid changes or cannabinoid receptor deficiency to the administration of exogenous cannabinoid (cannabis extract/cannabinoid constituent/cannabinoid agonist/antagonist). In this review, the effects of glial cell endocannabinoid signaling on cognition were impactful, in which the rodent models of AD showed preserved learning and memory. Cognitive recovery occurs following the administration of cannabinoids agonist. The endocannabinoid system, comprised of cannabinoid (CB: CB1 and CB2) receptors and their endogenous ligands, is engaged in various physiological tasks, most notably memory and learning. Numerous investigations have revealed that the endocannabinoid system strictly regulates cognition-related functions (Kruk-Slomka et al., 2017). The result from the analysis in the present review demonstrated that endocannabinoid-mediated glial cells exhibited considerable efficacy in shortening escape latency in pre-clinical AD models (*p* < 0.00001) and retaining recognition memory (*p* = 0.002). As the results of the recognition index and MWM tests were improved, experimental evidence indicates that this mechanism explains the beneficial effects of cannabinoids to stimulate endocannabinoid-mediated glial cells (Duffy et al., 2021).

Numerous memory processes, including consolidation, destabilization, and extinction, are highly influenced by the endocannabinoid system (Lunardi et al., 2020). Memory phase, brain site, and task-dependent manners may be affected by endocannabinoid-induced signaling pathways. Various experiments and clinical studies have shown that CB1R ligands affect memory and learning. Although CB1R ligands can both enhance and impair memory, each does so differently. The activation of cannabinoid CB1R in the basolateral amygdala (BLA) alleviated the memory impairment caused by scopolamine in adult male Wistar rats (Nedaei et al., 2016). The immediate footshock (context pre-exposure facilitation effect) and reversal learning, however, were inhibited by the hippocampal injection of CB1 antagonist (Lunardi et al., 2020). Such contradictory results could be attributed to the variations in behavioral tasks performed, handling techniques, the timing of drug delivery or the type of medication therapy, or other experimental settings, as well as doses and CB compounds chosen (Kruk-Slomka et al., 2017).

CB2R activation increases microglial cell migration and proliferation while reducing the release of inflammatory factors like TNF-α and free radicals, suggesting that anti-inflammatory effects occur secondary to activated CB2R in microglia (Rapaka et al., 2021). These effects also restore dendritic complexity in the cortex and improve memory for novel objects while not affecting plaque deposition or spatial memory (C. Li et al., 2019). CB2 agonists like MDA7 suppress microglial activation by Aβ fibril and facilitate Aβ disposal (Wu et al., 2013). CB2 knockout mice also exhibited better spatial memory in the Y-maze test (Y. Li and Kim, 2016). Memory and learning can be both facilitated and attenuated by CB2R ligands. These various memory effects may be primarily related to the pharmacokinetic properties of the CB2R ligands that have been tested and antioxidant properties that are indicated by both agonists and antagonists of these receptors (Kruk-Slomka et al., 2017). Overall, memory processes are modulated by CB1R and CB2R and they can be specifically targeted for pharmacological therapeutics to elicit the desired effects and prevent the undesirable ones once the roles of each kind of receptor are thoroughly characterized.

CB2R manipulation has been proposed to affect cognitive impairment because of its anti-inflammatory function and is commonly upregulated *via* microglial activity during neuroinflammation (Komorowska-Müller and Schmöle, 2021). As shown in our results, the implications of the pharmacological activation and genetic modification of CB2R in the AD mouse model are different in terms of how they affect the microglial activity and AD-induced neuroinflammation. While the pharmacological treatment requires CB2Rs to bind with molecules or ligands for activation to reduce the secretion of pro-inflammatory cytokines and improve cognitive behavior (Aso et al., 2013; Cheng et al., 2014; C; Li et al., 2019; Wu et al., 2013), CB2R deletion caused adverse effects (Galán-Ganga et al., 2021; Schmöle et al., 2015; Schmöle et al., 2018; J; Zhang and Chen, 2018). Moreover, differences in experimental designs may also have influenced the cognitive outcomes. We hypothesize that these could be attributed to the nature of CB2R itself, whether it is deficient, activated, or inactivated. When CB2R is deleted or antagonized, it mitigates inflammatory response, making it a neuroprotective factor. The same effect occurs when there is a presence of cannabinoid agonists. However, in progressing AD conditions, the inactivation or unoccupied of CB2R may lead to CB2 overexpression. This excess of CB2 during microglial activation would promote inflammatory cytokine release leading to neurodegeneration. Still, additional experiments are required to validate this hypothesis. Therefore, selective or non-selective cannabinoid agonists are crucial for microglial CB2R activation, with anti-inflammatory cytokine release to reverse the devastating ongoing inflammatory process in AD and overcome cognitive deterioration.

### 4.3 Glial cell-endocannabinoid-mediated synaptic plasticity

In synaptic plasticity, the downstream pathways of phosphoinositide 3-kinase (PI3K), phospholipase C-γ (PLCγ), and MAPK are activated as a result of BDNF’s high affinity binding to tropomyosin receptor kinase B (TrkB) (Guo et al., 2018). Depending on the route that is activated, the downstream physiological consequence of BDNF/Trk B activation followed by phosphorylation of CREB may involve modulation and enhancement of synaptic plasticity (De Vincenti et al., 2019), greater dendritic growth and branching (González-Gutiérrez et al., 2020), upregulation of diacylglycerol (DAG) synthesis, and promoted growth of neuronal fibers (Kowiański et al., 2018; Bergen, 2020). In the downstream regulation of synaptic signaling, endocannabinoid signaling appears to interact with BDNF activities. In neuroplasticity, the strength of synaptic signaling plays a significant role. The mechanisms of long-term depression (LTD) and LTP are particularly crucial to the neurobiology of memory and learning. Endocannabinoids play significant roles in controlling glutamatergic and GABAergic synaptic transmission as many retrograde messengers throughout the CNS (Dow-Edwards et al., 2017; Bergen, 2020).

In exploring endocannabinoid-mediated neurogenesis and neuroplasticity, Ferreira et al. discovered that CB1R selective activation and CB1R/CB2R non-selective activation increased cell proliferation and increased DG and subventricular zone (SVZ) cell proliferation, respectively. Regarding neuronal differentiation, both subtypes of cannabinoid receptors also enhanced neuronal differentiation in the DG and SVZ neurogenic niches (Ferreira et al., 2018). In the same study, Ferreira demonstrated that CREB is a key regulator of BDNF-induced gene expression and might also be the common linking element. In addition, De Chiara et al. uncovered a novel mechanism by which BDNF influences the function of striatal CB1R (De Chiara et al., 2010). In another study, Abd El-Rahman and Fayed (2022) reported improved cognition in D-galactose-injected ovariectomized rats *via* CB2R activation modulating the CREB/BDNF signaling pathway. This promoted CREB phosphorylation for the expression of various pro-survival genes such as BDNF and Bcl-2 which later enhanced the expression of both antioxidant enzymes and the antiapoptotic protein and reduced delayed neuronal death. Throughout neurogenesis, BDNF may be necessary for cannabinoid-induced effects on cellular proliferation and neuronal differentiation.

Glial cells play roles in several physiological processes, including programmed cell death, cell surveillance, the removal of newborn apoptotic neurons, neural plasticity, and synaptic pruning, among many others, that are essential for brain development and the maintenance of homeostasis in the adult brain (Sanchez-Varo et al., 2022). Neuroinflammation in AD driven by Aβ-induced neurotoxicity is alleviated by enhancing microglial endocannabinoid signaling. In the CA1 neurons of APP/PS1 transgenic mice, the LTP elicited by high-frequency electric stimulation was markedly impaired but restored with MDA7 treatment along with recovery of Sox (a neural stem cell marker) and decreased Iba1 marker and CB2 expression (Wu et al., 2017). Aso et al. (2016)reported that the administration of THC and CBD advanced stages altered the imbalance between excitatory and inhibitory neuronal activity in the somatosensory cortex of aged APP/PS1 mice, as evidenced by decreased GluR2/3 expression levels and increased GABA-A Rα1 expression. Via CB1R activation, THC and CBD both reduced the deleterious impact of Aβ on GABAergic function, which in turn improved cognitive function, facilitating inhibitory GABAergic activity in the somatosensory cortex despite the lack of changes in Iba1 and GFAP. A MAGL inhibitor instead of a cannabinoid agonist showed a decreased cognitive deficit with a reduction in astrocytic marker and CB2 expression in TG-CB2-KO mice treated with JZL184. The restoration of downregulated PSD95 and glutamate receptor subunit expression is likely linked to the improvement in cognitive function (J. Zhang and Chen, 2018).

### 4.4 Microglia-endocannabinoid mechanism on plaques aggregates

The results of the analysis in the present review demonstrated that endocannabinoid-modulating glial cells decreased amyloid plaques in the brain by enhancing amyloid clearance. Only one study in this review did not observe a decrease in beta-amyloid levels (Aso et al., 2012). Similarly, transgenic mice carrying a particular mutation in Beclin 1 (F121A) showed constitutively activated autophagy in the brain (among other tissues) and dramatically reduced amyloid accumulation (Rocchi et al., 2017). This may suggest that the agent promoting autophagy plays a pivotal role in plaque clearance and, thus, amelioration of cognition (Tamagno et al., 2018). Aβ may precipitate in brain tissues and trigger neuroinflammation by glial cells. Glial cells may also respond to endocannabinoid stimulation by activating upstream and downstream pathway cascades. These actions, which are required to ascertain the underlying neuroinflammatory mechanism, are regulated toward a safer mode without harming the surrounding neuron area.

Nevertheless, correlation analysis of the association between Aβ pathology and cognitive function showed that Aβ-42 was not significantly related to cognitive changes (El-Bakly et al., 2019). Instead of Aβ precipitation, glial cells directly affect behavioral function. The included studies showed that a decrease in glial cell activity was associated with the restoration of cognitive impairment in animals, as evidenced in the early and pre-symptomatic stages of AD when CB2R glial cell activation in APP/PS1 mice attenuated AD-dependent neuroinflammation (Aso et al., 2013). Although treatment with JWH-133 (Aso et al., 2013), THC/CBD (Aso et al., 2016), and JWH-015 (C. Li et al., 2019) decreased the release of pro-inflammatory cytokines, the Aβ plaque burden was unaffected. However, these treatments ameliorated cognitive performance in mice. Intriguingly, compared to the early symptomatic state, this impact was greater when the treatment was administered during the pre-symptomatic phase (Aso et al., 2013). Despite the association of CB2R overexpression with tauopathy in transgenic mice and patients with AD (Galán-Ganga et al., 2021), the amyloid burden causing CB2R overexpression was not correlated with cognitive status in humans (Solas et al., 2013). Consistent with a human study, Ma et al. (2021) reported that early Aβ accumulation had an independent effect on cognitive decline when mediated by either tau-related pathology, neurodegeneration, and neuroinflammation in subsequent cognitive decline in patients with mild cognitive impairment. Similarly, a cross-sectional study of 598 amyloid-positive participants found that participants with normal cognition and hippocampal volume were associated with preservation of high levels of soluble Aβ-42 despite increasing brain amyloidosis compared to those with mild cognitive impairment (MCI) and AD (Sturchio et al., 2021). Thus, the evidence showed that accompanying factors along with Aβ are required to induce cognitive deficits.

The findings from the current analysis suggest that the cognitive symptoms of AD may begin to appear upon the activation of neuro-immunological processes within the brain. Plaque accumulation does not immediately impair cognition but eventually precipitates the pathological process, symptom severity, and disease progression. Chronic treatment may be beneficial for attenuating the inflammatory state to augment cognitive function, which, in turn, promotes the clearance of protein precipitates. Thus, future studies should focus on tau pathology since brain hyperphosphorylated tau accumulation correlates more closely with cognitive decline than Aβ deposits (Johnson et al., 2016; Sanchez-Varo et al., 2022).

### 4.5 Microglia-endocannabinoid mechanism on neuroinflammation

Through toll-like receptor (TLR) activation, lipopolysaccharides (LPS) induce systemic inflammatory reactions. LPS binding triggers the activation of NF-κB to TLR4 on the microglia surface, which activates many signal transduction pathways, including MAPK, PI3K/AKT, and mTOR. Microglial activation mediates neuroinflammation following aberrant stimulation; tissue damage; and the presence of pathogens, infection or injury, and neurotoxins. Microglia will then aggregate, migrate, proliferate, phagocytose, present the antigen to T-cells, release various oxidants, and activate a variety of proteins and genes. Through NF-κB activation, microglia enhance the release of proinflammatory cytokines such as IL-1β, TNF-α, iNOS, reactive oxygen species (ROS), cyclooxygenase (COX)-1 and COX-2, and several neurotoxic agents, resulting in neuronal dysfunction and cell death. In chronic neuroinflammation, those cytokines and neurotoxic chemicals are released over a longer period, contributing to prolonged neurodegeneration.

CB1R/CB2R activation in the microglia recruits a variety of intracellular protein kinases that are implicated in the expression of various anti-inflammatory protein genes. Endocannabinoids inhibit MAPK pathway activity in activated microglia, leading to decreased IL-1β, IL-6, and TNF-α levels. In most of the selected studies, the endogenous cannabinoids AEA and 2-AG broadly modulated the immune system by increasing the production of anti-inflammatory cytokines such as IL-10 while decreasing the production of pro-inflammatory molecules *via* CB1R and CB2R activation in microglia cells (Krishnan and Chatterjee, 2012; Rapaka et al., 2021). CB2R is expressed by activated microglia in the brains of patients with AD and also in a similar model of dementia. The magnitude of reaction to partial and full cannabinoid agonists may be significantly influenced by receptor density. Cannabinoids elicit CB2R activation in microglia, which is responsible for their immunological effects. For example, CB2R activation inhibits the release of pro-inflammatory mediators, including TNF- α and free radicals, while promoting microglial cell proliferation and migration (Carrier et al., 2004; Dominicis et al., 2022). Therefore, activating CB2R in microglia might favorably affect inflammation. Additionally, the involvement of other functioning ‘endocannabinoidome’ receptors may contribute to the enhancement of the glial endocannabinoid signaling effects in AD (Cristino et al., 2019). Before initiating anti-inflammatory actions, CB2R activation by cannabinoid agonists establishing receptor-ligand interaction is assumed to further decrease CB2R expression, marked by the concurrent reduction of CB2 protein and microglial marker. Following this, the initial low CB1R expression during progressive and advanced AD may, in turn, become upregulated once the inflammation is resolved. However, additional experiments are required to validate this hypothesis. Our present work highlighted the reversal of microglia-mediated neuroinflammation following cannabinoids treatment (Marchalant et al., 2008; Martín-Moreno et al., 2012; Stumm et al., 2013; Aso et al., 2016; Wu et al., 2017) (Xiang et al., 2022) (C. Li et al., 2019) (Vázquez et al., 2015) (Schmöle et al., 2015) (Cheng et al., 2014) (Table 2).

### 4.6 Microglia-endocannabinoid mechanisms in immunomodulation

**FIGURE 11:**
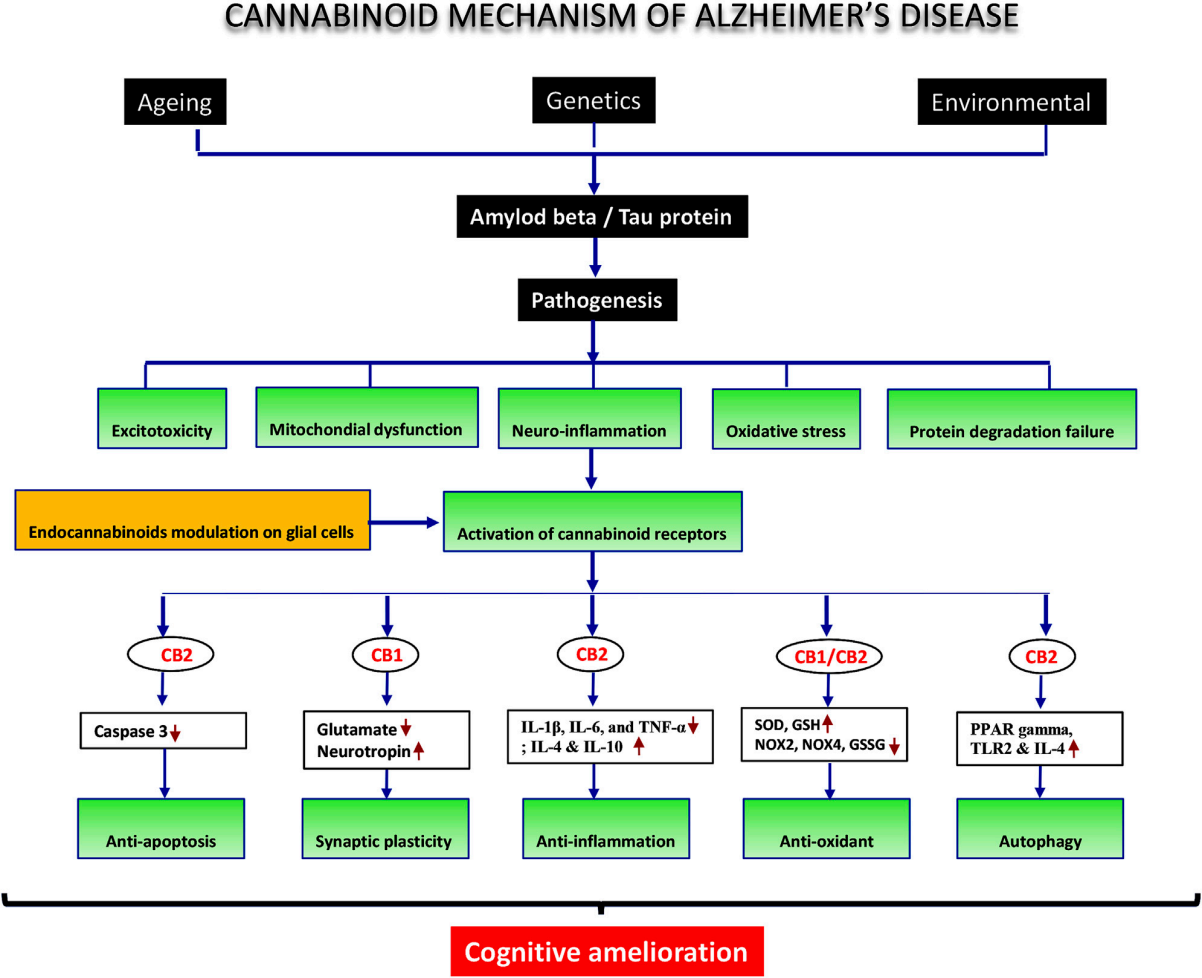
Schematic diagram of cannabinoid receptor activation and mechanism of action.

Our work revealed reduced immunoreactivity in glial cells, as evidenced by lower levels of GFAP (astrocytes) and Iba1 (microglia) in the cannabinoid-exposed animal model compared to the AD animal model. These reductions indicated that glial cells undergo polarization from A1/M1 to A2/M2 states. M2 microglia are an alternate microglia activation thought to be a phenotype of anti-inflammatory response, with neuroprotective effects and a role in tissue repair (Figure 11). Stimulation of interleukin-4/13 (IL-4)/IL-13) promoted M2 microglia predominance (Kwon and Koh, 2020; Tang and Le, 2016; Colonna and Butovsky, 2017). Markers of inflammation were dramatically upregulated in M1 microglia, while anti-inflammation factors were upregulated in M2 microglia of mice and rats (Lam et al., 2017).

The most evident phenotype of AD is cognitive and memory decline; thus, mitigating this dysfunction is the most crucial factor in determining how well potential medications are modeled and their treatment effectiveness. The attenuation of cognitive deficits in the transgenic AD model may occur due to the clearance of amyloid plaques, depending on the state of microglial polarization, which plays a critical role in this condition. The Aβ plaques in the alternative microglial states are believed to undergo either degradation by Aβ-degrading proteases (Saido and Leissring, 2012) or through autophagosomes for autophagy (Daily and Amer, 2020). In the brains of patients with AD, the latter process is more prevalent (Nilsson et al., 2013). As novel stimulation of memory and to augment the impact of systemic aspect on cognitive fitness, autophagy induction is not restricted to microglia but also occurs in hippocampal neurons to enhance activity-dependent functional and structural synaptic plasticity (Glatigny et al., 2019), astrocyte pruning of axons (Song et al., 2008), and increased myelination by oligodendrocytes (Smith et al., 2013).

CB2R activation induces phagocytosis (Mecha et al., 2015) and switches the M1 phenotype of microglial cells to the M2 phenotype (Orihuela et al., 2016). As M2 microglia upregulate endocannabinoid receptor expression, increased endocannabinoid release may, in turn, activate CB1R or CB2R and distinct endocannabinoids signaling cascades, intensifying the M2 profile (Mecha et al., 2016; Dominicis et al., 2022). The exposure of rat or human microglia to low concentrations of 2-AG and AEA increases the expression of the M2 marker Arg-1, Ym1/2, along with other markers of alternative phenotype such as suppressor of cytokine signaling 3 (SOCS 3) (Mecha et al., 2015). A range of endogenous, photogenic, and synthetic cannabinoids have been explored for their dual targeting of CB2R/PPARs to provide neuroprotective activities, mainly in rodent models of many neurodegenerative diseases (Lago-Fernandez et al., 2021). Ji et al. (2018) reported that antagonizing PPAR gamma promoted polarization from the M1 to the M2 phenotype in primary microglia, as indicated by increased M2 marker levels to enhance autophagy *via* LKB1–AMPK signaling and inhibited NF-κB–IKKβ activation. Toll-like receptor 2 (TLR2)-mediated autophagy may also regulate the M1/M2 phenotypic switch in BV2 microglial cells (Ma et al., 2020). The anti-inflammatory cytokine IL-4 causes M2 polarization of microglia and activates autophagic flux (Tang et al., 2019). The stimulation of autophagy may also cause microglial polarization into the M2 phenotype and suppress the inflammatory process (Zubova et al., 2022). Therefore, the selection of cannabinoid base medicine is pivotal for regulating microglia toward M2 polarization, enhancing the upstream signaling cascade of autophagy, and downregulating neuroinflammation.

### 4.7 Microglia-endocannabinoid mechanism in oxidative stress

The antioxidant and anti-inflammatory effects of phytocannabinoids are principally responsible for improving patient health, even though there remains much to learn about cannabis-based medicine (CBM)-mediated modulation of the ECS (Maayah et al., 2020). The so-called endocannabinoidome, which is an ECS expansion to incorporate the receptors, enzymes, and second messengers under cannabinoids regulation, has diverse targets for these phytocannabinoids (Ligresti et al., 2016). In addition to CB1 and CB2, other receptors respond to these molecules, including the transient receptor potential cation channel subfamily M member 8 (TRPM8) and transient receptor potential channels of vanilloid type-1 (TRPV1) (De Petrocellis et al., 2007); the orphan G-protein-coupled receptors (GPCR) 55, 18, and 119 (GPR55, GPR18, and GPR119); and peroxisome proliferator-activated receptor gamma (PPAR-α) (Cristino et al., 2019).

Emerging evidence suggests that ECS can modify the expression and/or activity of enzymes implicated in the formation of these small reactive molecules such as NADPH oxidase enzymes 2 and 4 (NOX2 and NOX4) and also control cellular ROS/RNS generation by regulating mitochondrial-derived ROS/RNS (Lipina & Hundal, 2016). CB1 activation increases redox imbalance, whereas CB2 stimulation reduces ROS/RNS production (Han et al., 2009). By increasing intracellular SOD and GSH levels, lowering the oxidized glutathione (GSSG) level, raising the GSH/GSSG ratio, and reducing NOX2 expression, AEA protected a mouse hippocampal neuron cell line against H_2_O_2_-induced redox imbalance. These effects were nullified by the administration of a CB1 antagonist or CB1-siRNA, indicating the ability of AEA to reduce oxidative stress in hippocampal neurons that might be mediated by CB1 activation (Jia et al., 2014). Moreover, arachidonyl-2-chloroethylamide activates mitochondrial CB1, reducing oxidative stress and exerting neuroprotective effects on I/R damage (Paloczi et al., 2018). CB2 activation seems to play a role in mitigating I/R damage by reducing ROS/RNS generation and lipid peroxidation (Paloczi et al., 2018). Palmitoylethanolamide (PEA), dependent on PPAR-α, protects glia from oxidative stress by decreasing MDA production (Scuderi et al., 2018). TRPV1 is involved in vascular dementia once activated by AEA, which reduces oxidative stress, improves learning and memory, and enhances neuroprotection (Gallelli et al., 2018). Additionally, N-arachidonoyldopamine (NADA), an endogenous ligand of CB1, TRPV1, and PPAR-γ, belongs to the endovanilloid class of endocannabinoids and has anti-inflammatory and antioxidant effects on glial cells.

Our findings indicated that cannabinoids play roles as antioxidants by activating GPR55, where acute administration of O-1602 elevated SOD and depressed MDA with concomitant reduced microglial activation (Xiang et al., 2022). Chronic daily cannabinoid treatment with JWH-133 also ameliorated cognitive symptoms in an experimental model of AD by reducing inflammation, oxidative stress, and microgliosis *via* CB2R activation (Aso et al., 2013). Similarly, reduced astrocyte activation and lipid peroxidation were observed in chronic EFC treatment (Chen et al., 2017). The decreased MDA levels and increased SOD activities in the rat brains following WIN administration could be attributed to the positive effects of WIN on maintaining redox balance as well as their interactions with various signaling pathways that orchestrate neuronal survival, differentiation, and death (Mahdi et al., 2021).

### 4.8 Endocannabinoid-mediated astrocyte signaling

The different mechanisms by which astrocytes interact with other cell types are essentially controlled by intracellular Ca^2+^ concentrations. Astrocytic CB1 responds to endocannabinoids generated during neuronal activity by eliciting increased Ca^2+^ levels, as evidenced by astrocytic-neuron communication in synaptic physiology (Bernal-Chico et al., 2022). As a general and fundamental mode of endocannabinoid signaling in the modulation of astrocyte activity, astrocyte Ca^2+^ mobilization *via* CB1R is thought to occur in the cortex and hippocampal human tissue and throughout the rat brain. Astrocytic CB1 activation causes increased cytosolic Ca^2+^ levels, which helps to release gliotransmitters like glutamate and D-serine and indirectly facilitate excitatory transmission (Han et al., 2012; Mahmoud et al., 2019; Robin et al., 2018). This discovery underscores the importance of CB1R in the modulation of synaptic activity by astrocytic cells while also emphasizing the unique role of astroglial CB1 in the effects of cannabis-based medications in humans. Despite a few studies that expressed CB2R in astrocytic cells, there is currently a lack of strong evidence for the existence of astroglial CB2R under physiological settings. Surprisingly, one of the studies included in this review reported reduced GFAP in APP transgenic mice lacking CB2R following 3 weeks of JZL184 administration (Zhang & Chen, 2018). In that study, GFAP was used instead of Iba1 to assess its association with CB2R. The CB2R deficiency might have resulted in reduced microglia activation, which may also indirectly influence astrocyte action, represented by decreased GFAP, which is required for the close relationship between CB2R and astrocyte compared to microglia.

Glial cells regulate not only aggregated protein clearance but also the anti-neuroinflammatory, immunomodulation, redox stability, and eventually neuroplasticity causing amelioration of learning and memory, which are key mechanisms occurring with endocannabinoid modulation. As with microglia, the astrocyte react to brain damage or neurodegeneration as an adaptive mechanism in response to injury or illness. Astrocytes undergo reactive astrogliosis in pathological conditions such as the accumulation of Aβ and pathogenic tau. The re-expression of nestin and the upregulation of glial fibrillary acid protein (GFAP) and vimentin in the above conditions are considered markers of astrocyte reactivity (Sanchez-Varo et al., 2022). Mahdi et al. reported that increased nestin levels might affect stem cell migration and differentiation, as evidenced by improved cellular activity in the brain, GFAP levels, and cognitive status following treatment with WIN or donepezil in AlCl_3_ and D-galactose-induced AD rats (Mahdi et al., 2021).

Overall, the present works showed that cannabinoid agonists decreased astrocyte activation. In MAGL-deficient mice, Chen et al. (2012) reported that decreased GFAP with MAGL inhibition was connected to greater hippocampus LTP and enhanced learning and memory *via* a CB1 receptor-dependent mechanism. Reducing astrocyte activation reduced oxidative stress after daily EFC treatment for 14 weeks in D-galactose-induced AD rats (Chen et al., 2017). Wu et al. (2013)reported that chronic MDA7 administration promoted Aβ clearance, ameliorated Aβ-induced glia activation and production of IL-1β, and restored CB2 expression with subsequent synaptic plasticity, memory, and cognition in Aβ1–40 fibrils injected in the hippocampus. Later, Wu et al. (2017)showed that using the same agents with longer duration showed the same results in APP/PS1 mice. Sox expression was recovered, suggesting that MDA7-mediated microglia CB2R activation rescued neurogenesis and improved cognition in the AD mouse model. A low extent of astroglial activation should be aligned with CB1 activation. However, the corresponding cognitive deficits in APP23/CB1−/− mice showed decreased astroglial marker and also reduced sAPPa, its C-terminal a and b fragments, and Aβ1-40 peptide in the brain (Stumm et al., 2013), which might reflect a lower amyloid plaque load.

### 4.9 Endocannabinoid modulation on astrocytes-microglial communication

Our work has accumulated evidence on the mutual response of microglia and astrocytes. Most of the articles evaluating both microglial and astrocytes reported increased activity of both in neuroinflammation and decreased reactivity in neuroprotection. Compelling evidence suggests synchronization and communication between microglia and astrocytes in healthy and diseased brains (Vainchtein & Molofsky, 2020). Microglia, in particular *via* nuclear factor-B (NF-κB) signaling, may potentiate the inflammatory activation of astrocytes by elevating cytokine and chemokine expression levels (Kirkley et al., 2017). Cytokines (TNF-α, IL-1, and C1q) released by microglia may alter the supportive function of astroglia (Liddelow et al., 2017). In an *in vitro* cell culture study, Kim et al. (2021) showed that astrocyte-microglia cross-talk is advantageous for microglia proliferation with M2 type acquisition and A2 type polarization of astrocytes. In AD mouse models where the complement system was engaged, the interaction between the complement factor C3 produced by astrocytes and the microglial C3a receptor (C3aR) controlled the dynamic regulation of microglial phagocytosis (Lian et al., 2016).

Microglia and astrocyte interactions are also observed. Aso et al. conducted two stages of initiation of cannabinoid treatment in APP/PS1, in which the presymptomatic stage showed restoration of the long-term memory decline. However, they reported no appreciable changes in aversive avoidance learning capacity when therapy was initiated at the symptomatic stage. Despite no significant decrease in Aβ plaques, the GSK3, p38, and SAPK/JNK kinase activity may have decreased, which would explain the decline in tau hyperphosphorylation level, establishing a role of CB2R in GSKβ modulation (Aso et al., 2013). The combination of THC and CBD reduced soluble Aβ42, but not Aβ40 protein levels, thus lowering the levels of the most hazardous soluble Aβ form in APP/PS1 animals to provide a protective effect and increase the expression of thioredoxin 2 and Wnt16 to contribute to axonal integrity (Aso et al., 2015). Through CB1R activation, the above combination also supports inhibitory GABAergic activity in the somatosensory cortex by reducing the negative impact of A on GABAergic function and, ultimately, on cognitive performance. In addition, chronic stimulation of CB1R significantly suppresses glutamatergic activity, which helps to enhance cognitive performance by reversing the alterations in neuronal excitability observed in APP/PS1 animals (Aso et al., 2016). The ubiquitous distribution of the endocannabinoid system and its multifunctionality suggest that the favorable cognitive effects reported in APP/PS1 following chronic treatment with natural cannabinoids may result from parallel mechanisms.

### 4.10 Relationships between animal and clinical research

Cannabinoids have recently been studied in several fields, including neuroscience. Although pre-clinical research has produced compelling results, some clinical trials have also reported potentially positive outcomes. A systematic review and meta-analysis found strong evidence of the effectiveness of cannabinoids for the treatment of dementia-related neuropsychiatric symptoms (Bahji et al., 2020). No randomized controlled trials (RCTs) on the use of cannabinoids to treat cognitive deficits in dementia have yet been conducted, but a systematic review of RCTs on the effectiveness of cannabinoids for treating dementia showed improvement in the behavioral and psychological symptoms of dementia (BPSD) for nabilone over THC (Charernboon et al., 2021). A systematic review of human studies reported mixed findings on the effects of ECS on cognition in AD in investigations range from epigenetic to imaging and blood and cerebrospinal fluid studies (Berry et al., 2020). The major challenges of human studies compared to animal studies are the lack of an ability to sample the brain tissues of humans. Thus, glial cells cannot be sampled in living human brains except when there is a strong indication for surgical intervention. Therefore, the current practical method of obtaining human brain samples through post-mortem examinations, in which data from a comprehensive review demonstrated the association of endocannabinoids and glial cells in AD (Berry et al., 2020). Although complete tissue findings have been reported, the lack of simultaneous cognitive function is a notable shortcoming of post-mortem samples. Thus, animal studies should reflect complementary work in humans. The exploitation of appropriate investigative modalities such as AD-specific plasma biomarkers for screening (Cullen et al., 2021) and performing positron emission tomography (PET) scans may provide molecular imaging to potentially unveil diagnostic CB receptors (Ahmad et al., 2014; Ahmad et al., 2016) and glial cell changes (Edison et al., 2018; Cavaliere et al., 2020) to identify subtle findings in AD research in humans.

## 5 Limitations

This review has several limitations. Since only a limited number of studies were included, only an evidence-backed basis for subsequent experiments through the systematic review of some of these potential mechanisms was provided. Moreover, the meta-analysis showed high heterogeneity in certain parameters, which frequently occurs in animal studies due to differences in experimental methods. The high proportion of unclear risk of bias (Figure 10) resulted from inadequate information regarding the assessment of certain types of bias domains reported by the authors in a primary study without knowing whether they had assessed a particular type of bias. Therefore, the risk of bias analysis in primary studies should be improved by encouraging researchers conducting animal studies to comply with the recently revised ARRIVE guidelines 2.0 (du Sert et al., 2020). These guidelines ensure a standard of reporting by prompting authors and journals to identify the minimal information required to report in publications describing animal experiments to allow accurate and transparent reporting.

## 6 Future therapeutic perspectives

Based on the data of the included studies analyzing the pharmacological properties of selective CB1R, CB2R, or non-selective cannabinoid agonists, we speculate that non-selective cannabinoid agonists might offer a better outcome due to their flexibility on either CB1 or CB2 receptors, which exert different mechanisms. CB1R provides cognitive improvement against synaptic dysfunction while CB2R provides neuroprotection against inflammatory responses. However, additional research is required to validate this speculation. The classification of CB1R and CB2R agonists and their respective pharmacodynamic properties according to the abovementioned mechanism may provide information to allow more precise targeting in therapeutic strategies for AD. The communication between microglia and astrocytes is also complex in the context of achieving the treatment aim of preserving cognitive functions. The analysis results indicated that pharmacological manipulation of endocannabinoid system-mediated glial cells through the intake of cannabinoid agonists may be a candidate for the clinical treatment of AD. The present study summarized and discussed the possible pharmacological mechanisms involved and also addressed the importance of the effects of endocannabinoids on symptomatology-dependent immunomodulation as a primary treatment target in AD combined with anti-amyloidogenic agents as secondary and complementary therapies.

## 7 Conclusion

To our knowledge, this is the first systematic review to integrate the results of ECS-linked glial cell changes in AD from all known animal research studies published in the last 17 years. ECS signaling that directly shifts microglial morphology into the neuroprotective (M2) and homeostatic (M0) phenotype would be the main events toward a major outcome of cognitive improvement, in which synaptic plasticity modulation, synaptic pruning, and neural trophic support are considered the crucial physiological roles of microglia. We postulate that the ECS effects from the administration of cannabinoid agonists lead to endocannabinoid modulation of glial cells, particularly CB2R activation, to influence the mechanistic sequence as follows: 1) glial cell phenotype transformation toward the alternative state, 2) increased anti-inflammatory cytokine and reduced pro-inflammatory cytokine levels, 3) promotion of glial cell autophagy for clearance of protein aggregation, 4) decreased ROS/RNS generation and lipid peroxidation with increased antioxidant levels, and 5) amelioration and maintenance of synaptic plasticity. The findings support the view that changes in CB1R, CB2R, central AEA concentration, FAAH, and MAGL activity occur in AD animals despite some methodologically heterogeneous data. In general, the findings in this review provide knowledge to establish ECS biomarkers in AD and may offer opportunities for the development of novel drugs.

In conclusion, CB2R in glial cell activation is the rate-limiting step before endocannabinoids exert their neuroprotective effects. Additionally, the synergistic involvement of CB1R is required to amplify the positive neurocognitive impact. As the endocannabinoid system is a near-ubiquitous regulator of neuronal communication throughout the brain, studies involving transcriptional and epigenetic mechanisms are needed to elucidate endocannabinoid-related synaptic plasticity.

## Data Availability

The original contributions presented in the study are included in the article/Supplementary Material. Further inquiries can be directed to the corresponding authors.
