# Supplementary material for: Regulatory role of the endocannabinoid system on glial cells toward cognitive function in Alzheimer’s disease: A systematic review and meta-analysis of animal studies
Source: Front Pharmacol. 2023 Mar 3;14:1053680. doi: 10.3389/fphar.2023.1053680 (PMC10028478; doi:10.3389/fphar.2023.1053680)
Supplement: Supplementary file 1 [file Table1.pdf]

Supplementary Table 1: List of 6 articles excluded from the study selection process

Authors	Titles	Source	DOI
Bajaj et al 2022	Effect of the MAGL/FAAH Dual Inhibitor JZL-195 on Streptozotocin-Induced Alzheimer's Disease-like Sporadic Dementia in Mice with an Emphasis on Aβ, HSP-70, Neuroinflammation, and Oxidative Stress.	ACS Chemical Neuroscience	10.1021/acschemneuro.1c00699
Gordon et al. (2022)	Analysis of the Effect of Neuroprotectors That Reduce the Level of Degeneration of Neurons in the Rat Hippocampus Caused by Administration of Beta-Amyloid Peptide Aβ25-35	Bulletin of Experimental Biology and Medicine	10.1007/s10517-022-05410-9
Memudu et al. (2021)	An investigative study to demonstrate the link between combined intake of marijuana and codeine in Alzheimer's disease pathology.	Alzheimer's & dementia: the journal of the Alzheimer's Association	10.1002/alz.058465
Sudeep et al. (2021)	A standardized black pepper seed extract containing β-caryophyllene improves cognitive function in scopolamine-induced amnesia model mice via regulation of brain-derived neurotrophic factor and MAPK proteins.	Journal of Food Biochemistry	10.1111/jfbc.13994
Kanojia et al. (2021)	Beta-Caryophyllene, a CB2R Selective Agonist, Protects Against Cognitive Impairment Caused by Neuro-inflammation and Not in Dementia Due to Ageing Induced by Mitochondrial Dysfunction.	CNS and Neurological Disorders - Drug Targets	10.2174/1871527320666210202121103
Lunardelli et al. (2019)	Co-ultraPEALut: Role in preclinical and clinical delirium manifestations.	CNS and Neurological Disorders - Drug Targets	10.2174/1871527318666190617162041
