# Supplementary material for: Regulatory role of the endocannabinoid system on glial cells toward cognitive function in Alzheimer’s disease: A systematic review and meta-analysis of animal studies
Source: Front Pharmacol. 2023 Mar 3;14:1053680. doi: 10.3389/fphar.2023.1053680 (PMC10028478; doi:10.3389/fphar.2023.1053680)
Supplement: Supplementary file 2 [file Table2.pdf]

Supplementary Table 2: The pathogenesis of cognitive impairment in rodent and the possible mechanism of glial cell endocannabinoid signaling

Authors	Pathogenesis of cognitive impairment	Possible Mechanism of Glia cell - Endocannabinoid Signaling
(Abd El-Rahman & Fayed, 2022)	• Estrogen depletion (induced by bilateral ovariectomy of female rats) substantially contributes to memory loss and cognitive decline.<br>• D-gal at high doses bounded with the free amino groups of proteins to produce advanced glycation end products (AGEs) inducing oxidative stress and TLR4 expression.	• Activation of CB2R via CB2R agonist (AM1241)<br>○ Reduces pro-inflammatory cytokines (IL-6, IL-12, and TNF-α) via mitigation of TLR4 expression, signaling and its downstream effector NF-kb which subsequently alleviate spatial and recognition memory impairments.<br>○ Inhibited TLR4 expression causing shifting the M1 to M2 state of glial cell and<br>○ Modulates CREB/BDNF signaling pathway → Phosphorylation of CREB enhances the expression of various pro-survival genes as Bcl-2 and BDNF.
(Xiang et al., 2022)	• Intracerebroventricular streptozotocin (i.c.v-STZ) injection:<br>○ Produces cognitive deficits in mice, as well as the accumulation of Aβ, hyperphosphorylation of tau, cholinergic deficiency, and synaptic dysfunction.<br>○ Cause neurons brain to have high rates of energy production and oxygen consumption, making them extremely sensitive to the overproduction of reactive oxygen species and oxidative damage.	• O-1602 treatment:<br>○ Reversed STZ induced GPR55 down-regulation, reduced the activity of β-secretase 1 (BACE1) and the level of Aβ1–42, and abolished the up-regulation of acetylcholinesterase (AChE) activity in the hippocampus and frontal cortex.<br>○ Suppressed STZ-induced oxidative stress, as well as attenuated neuroinflammation as indicated by decreased series of pro-inflammatory cytokines and microglia activation.<br>○ Ameliorated synaptic dysfunction by promoting the up-regulation of PSD-95 protein in the STZ-treated mice.
(Galán-Ganga et al., 2021)	• hTAUP301S induced selective neuronal CB2 expression in a late stage tauopathy mouse model.<br>• Cnr2+/+ mice, hTAUP301L overexpression significantly decreased BDNF mRNA levels.<br>• Cnr2+/+ mice injected with the AAV-hTAU P301L in the hippocampus showed the expected significant decrease in the discrimination index.	• CB2 receptors have been shown to regulate a plethora of kinases, including PI3K/AKT/GSK-3, JNK and p38, which are linked to TAU phosphorylation.<br>• Therefore, lack of CB2 seen in CB2 deficient mice (Cnr2-/-) could be implicated in reducing TAU phosphorylation → improves the cognitive impairment and the synaptic plasticity induced by hTAUP301L overexpression.
(Mahdi et al., 2021)	• AlCl3 and D-gal induced rats' model:<br>○ Impaired cognitive function, Glial cell activity<br>○ Decreased the SOD and GSH levels	• WIN55,212-2 treatment:<br>○ Reversed oxidative stress by reducing the levels of MDA and increasing the levels of SOD and GSH<br>○ It elevates the levels of neurogenesis biomarkers, GFAP and Nestin, which is evident by a high number of viable neurons in the hippocampus.
(Li et al., 2019)	• APP/PS1 mice:<br>○ Increased the microglial activity<br>○ Highly expressed TNF -α expressions causing cognitive impairment	• JWH-015 treatment:<br>○ CB2R activation prevented NOR dysfunction but did not affect the spatial cognitive impairment.<br>○ Reduced microglial immunoreactivity, promoted M1/M2 microglial phenotype conversion<br>○ Activation of CB2R effectively reduced the immunoreactivity of Iba-1.<br>○ Promoted M1/M2 microglia conversion in the cortex but not hippocampus, enhancing mRNA expression of M2 microglia biomarkers Ym1/2
(Patricio-Martínez et al., 2019)	• Aβ (35 -25) injected mice:<br>○ Highly expressed beta amyloid depositions.	• Aβ (35 -25) with ACEA treatment:<br>○ Activation of CB1R prevents memory loss, iNOS expression and consequently decreases NO<br>○ Prevents the neurodegeneration in the CA1 region of the hippocampus.

(Zhang & Chen, 2018)	• TG-CB2R-KO:<br>o Increases Expression of BACE1 and Production of Aβ.<br>o Promote Astrocytic Reactivity and Reduces Neurodegeneration.<br>o Prevent 2-AG Metabolism with enhancing Deterioration in Expression of Synaptic Proteins	• TG-CB2-KO mice with inactivation of MAGL is likely associated with:<br>o Restoration of downregulated expression of PSD95 and glutamate receptor subunits.<br>o 2-AG are likely mediated by activation of PPARγ via CB1/2-depednet and/or -independent pathways<br>o PPARγ displays significant anti-inflammatory properties through suppression of NF-κB.
(Schmöle et al., 2018)	• APP/PS1 mice:<br>o Gene expression for receptors TLR4 and ager were significantly enhanced.<br>o Gene expression for receptors for amyloid degrading enzyme decrease.<br>o Aβ accumulation have been linked to deficits in spatial learning and memory.	• APP/PS1*CB2 −/− mice:<br>o Reduced plaque loads → enhanced expression of APP secretases<br>o Plaques were smaller and more condensed than APP/PS1 mice<br>o Inhibit microglia and macrophage activation and therefore promotes a reduction in neuroinflammation (microglia become more ramified).<br>o NeuN+ increased
(Aparicio et al., 2018)	• 5xFAD/FAAH−/−:<br>o Increase in the M1 to M2 ratio of microglia.<br>o Increase IL1β and TNFα while decrease IL10 and IL4.	• 5xFAD/FAAH−/− mice treated with minocycline mice:<br>o IL1β mRNA levels were normalized by inhibition of IL1β synthesis.<br>o Decrease in amyloid plaque and Aβ1-42 levels.
(N. Y. Chen et al., 2017)	• Intraperitoneal injection of D-gal:<br>o Severe accelerated aging and induction of memory impairment<br>o Increased MDA levels and SOD activities.<br>o Increased neuroinflammatory process (raised levels of GFAP).<br>o Elevation of hippocampal hyperphosphorylated tau and expression of presenelin 1 were also detected.	• EFC treatment in D-gal-rats:<br>o Provided and anti-oxidization property that raises SOD activity and reduces MDA levels<br>o Alleviated the cognitive deficits caused by D-gal treatment, in part by inhibiting inflammatory reactions (reduce the levels of GFAP) in the brain.<br>o Lower tau phosphorylation via attenuating tau phosphorylation<br>o Reducing tau phosphorylation and inhibiting PS1 expression.<br>o Interfering with Notch 1 signaling and promoting pathogenic alterations in tau, PS1 overexpression.
(Wu et al., 2017)	• APP/PS1 mice:<br>o Promote glial cell immunoreactivity and CB2R expressions in CA1 hippocampus, DG and entorhinal cortex.<br>o Aggravated Aβ plaques accumulation.<br>o Reduction in Sox2 expression.	• APP/PS1 mice with MDA treatment:<br>o Treatment with a CB2 agonist significantly reduces microglial activity (Iba-1)<br>o The CB2 receptor functions as a negative feedback regulator and activating it with a CB2 agonist can decrease the severity of the neuroinflammatory response and the subsequent development of neuronal injury in the CNS.<br>o Restored Sox2 immunoreactivity in the DG of APP/PS1 mice, rescuing neurogenesis.
(Aso et al., 2016)	• APP/PS1 transgenic mice model:<br>o The amyloid deposition in the mouse line advances with ageing and in a region-specific manner, with a corresponding rise in microglial activation and astrogliosis, as well as cognitive impairment and decreased LTP.	• Chronic THC treatment:<br>o Restored presynaptic SNAP25 but not postsynaptic PSD-95 protein levels.<br>o Through activation of CB1 receptors, it promotes inhibitory GABAergic activity in the sensorimotor cortex by minimizing the harmful effect of A-beta on GABAergic function.<br>o The decreased glutamatergic activity brought on by repeated activation of the CB1 receptor.

(Vázquez et al., 2015)	• 5xFAD/FAAH/mice<br>○ The astrocytes lacking fatty acid amide hydrolase (FAAH) activity exhibited an exacerbated inflammatory response that was especially evident when challenged with a proinflammatory stimulus, such as beta-amyloid.<br>○ FAAH leads to deficit in anandamide (AEA) production and promote an increase in pro-inflammatory phenotype particularly involving microglial cells towards cognitive dysfunction in AD.	• 5xFAD/FAAH-/- mice:<br>○ Significant reduction in plaque-associated astrogliosis and microgliosis by using GFAP and Iba1 as markers.<br>○ Alters the production of amyloid peptides which includes significant decreases in the amount of total APP, soluble Ab1e40, and Ab1e42 peptides and neuritic plaque density.<br>○ Microgliosis and astrogliosis were also diminished.
(Schmöle et al., 2015)	○ APP/PS1 mice:<br>○ Increase of ICAM-1 and CD40, IL-6, TNFα, and CCL2<br>○ Amyloid-b processing and plaque deposition are increase<br>○ Microgliosis	• APP/PS1*CB2-/- mice:<br>○ TNFα and CCL2 expression levels are reduced.<br>○ Decrease microgliosis due to CCL2 expression → phagocytic uptake of Aβ by CB2-/- microglia.<br>○ Reduced brain levels of soluble Ab 40/42 but equivalent Ab plaque load.
(Aso et al., 2015)	○ APP/PS1 mice:<br>○ Aβ40 and Aβ42 burden in the cortex.<br>○ Increase number of astrocytes around the plaques.<br>○ Mapk3, Psmb2, Txn2, and Wnt16 expression increased	• APP/PS1 mice treated with THC, CBD, or the combination of both:<br>○ A reduction of the astrogliosis associated with Aβ deposition in Aβ APP/PS1 mice<br>○ Significantly reduced microgliosis and the expression of several cytokines and related 687 molecules in Aβ PP/PS1 mice.<br>○ The activation of CB1 receptor in vitro preserves neuron viability by reducing Aβ induced lysosomal membrane permeability.<br>○ CB2 receptor agonists induce Aβ removal by human macrophages and reduce microglial response to Aβ.
Cheng et al., 2014)	• APP/PS1 mice:<br>○ Elevated Aβ burden.<br>○ GFAP upregulation when astrocytes are activated.<br>○ Increased Iba1 expression.<br>○ mRNA levels of the other two proinflammatory cytokines, TNF-α and IL-1β, increased.	• APP/PS1 mice with β-Caryophyllene treatment:<br>○ Reduced β-amyloid plaque burden, reduced numbers of activated microglia, and lower levels of inflammatory markers.<br>○ Involves both CB2 receptor activation and the PPARγ pathway.<br>○ Exhibits long-lasting anti-inflammatory properties in different inflammatory models.
(Stumm et al., 2013)	• APP23 mice:<br>○ Numerous plaques were found in the cerebral cortex and in the hippocampal area.<br>○ Extensive glial activation in close vicinity to amyloid plaques in the neocortex.<br>○ Thinning of hippocampal CA1 region	• APP23/CB1-/- mice:<br>○ Plaque number and astrogliosis were significantly reduced. Glial cell activation reduced was a reflection of reduced amyloid plaque load. altered processing of APP, which results in a reduced number of APP-cleavage fragments and, in consequence, also in a reduced number of amyloid plaques.<br>○ Deterioration in the cognition tests<br>○ Mutant APP overexpression and CB1 knockout causes an early excitotoxicity and provokes seizures, which may eventually lead to premature death in most of the APP23/CB1-/- animals.

(Wu et al., 2013)	• Aβ1– 40 mice:<br>○ Enhanced Aβ1–40-induced glia activation in the hippocampal CA1 area.<br>○ Induced CB2 receptor upregulation.<br>○ Increased IL-1β protein expression.<br>○ Aβ1– 40 accumulations.<br>○ Impaired glutamatergic transmission	▪ Aβ1– 40 mice with MDA7:<br>○ Activation of central microglial CB2 receptors.<br>○ Enhances glia activation which induces IL-1 production.<br>○ Restoration of synaptic plasticity, cognition, and memory.
(Aso et al., 2013)	• APP/PS1 mice:<br>○ CB2 gene expression.<br>○ Aggravated the cortical and hippocampal Aβ burden, the cortical soluble fraction, or synaptic markers.<br>○ Enhanced tau phosphorylation and selected tau kinase activity.<br>○ Promoted gliosis associated with Aβ deposition and cytokine expression.	▪ APP/PS1 mice with JWH-133 treatment:<br>○ Decreased microglial reactivity and reduced expression of pro-inflammatory cytokines IL-1, IL-6, TNF, and IFN.<br>○ Decreased tau phosphorylation and selected tau kinase activity in the vicinity of Aβ plaques.<br>○ Reduction of oxidative stress damage.
(R. Chen et al., 2012)	• 5XFAD mice:<br>○ Monoacylglycerol lipase (MAGL) is the primary enzyme metabolizing the endocannabinoid 2-arachidonoylglycerol in the brain.<br>○ Impaired the integrity of Hippocampal Synaptic Structure.	▪ Inactivation of MAGL:<br>○ Suppressed production and accumulation of β-amyloid (Ab) associated with reduced expression of β -site amyloid precursor protein cleaving enzyme 1 (BACE1) in a mouse model of AD.<br>○ Prevented neuroinflammation, decreased neurodegeneration, maintained integrity of hippocampal synaptic structure and function, and improved long-term synaptic plasticity, spatial learning, and memory in AD animals.
(Fakhfouri et al., 2012)	• Aβ (1-42) mice:<br>○ decrease in both expression and transcriptional activity of PPAR-ϒ.<br>○ Aβ-induced expression of active caspase 3 accompanied by the appearance of TUNEL-positive neurons in CA1 subfield, indicative of apoptotic cell death.	• Aβ (1-42) with WIN treatment:<br>○ WIN enhanced the Ab-triggered induction of PPAR-ϒ transcriptional activity. Activation of cannabinoid CB1 receptor by WIN contributes in part to the induction of PPAR- ϒ activity.<br>○ cannabinoid receptor as well as PPAR-g-mediated downregulation of the inflammatory signaling events associated with Ab exposure.<br>○ prevented NF-kB migration to the nucleus mediated by WIN on CB1 and CB2 against NF-kB.
(Martín-moreno et al., 2012)	• TgAPP+Veh:<br>○ reactive astrocytes in proximity with Aβ plaques.<br>○ COX-2 protein levels and TNF-a mRNA expression.<br>○ increased tau phosphorylation, mainly by the action of GSK3-β.	• TgAPP+WIN/JWH:<br>○ Prolonged oral JWH treatment decreased Aβ1-40 levels in brain and both cannabinoids.<br>○ decreased the more amyloidogenic fragment, Aβ1-42 by favoring Aβ transport.<br>○ reduce microglial activation and inflammatory parameters (COX2).
(Aso et al., 2012)	• AβPP/PS1:<br>○ exhibit hyperphosphorylated tau protein in the vicinity of Aβ plaques.<br>○ Progressive age-dependent loss of CB1 receptor in the neocortex of AβPP/PS1 mice at advanced stage.<br>○ Hypertrophic astrocytes and reactive microglia were observed in the vicinity of Aβ plaque.	• AβPP/PS1-ACEA:<br>○ Reduction of the astrocytic responses associated with Aβ deposition.<br>○ reduced the GSK3β phosphorylation at Ser9 induced by Aβ in vitro and in vivo.<br>○ stimulation of CB1 receptor activates the pro-survival PI3K/Akt pathway, leading to the inactivation of GSK3β by phosphorylation at Ser9 promotes protection against excitotoxicity induced by Aβ.<br>○ Reduced tau phosphorylation at Thr181 in the area surrounding Aβ deposition.<br>○ reduction in the expression of the pro-inflammatory cytokine IFN-ϒ in astrocytes.

(Martín-Moreno et al., 2011)	• Mice injected with Aβ:<br>• extensive clustering of activated microglia at sites of Aβ deposition in AD brain.<br>• TNF-α and IL-6 expression were markedly increased.<br>• Nitric oxide (NO) was increase.	• Cannabinoid agonist (CBD/JWH):<br>• Inhibit the intracellular calcium increase brought about by high concentrations of ATP in microglial cells may be mediated by cannabinoid or A2A receptors.<br>• Mediate CB2 receptors in migration of N13 and primary microglial cells.<br>• Increase endocannabinoid availability, such as the inhibitor of endocannabinoid (VDM11) reuptake to prevent Aβ -induced cognitive deficits.<br>• Abolished IL-6 and TNF-α expression increase mediated by microglia.
(Marchalant et al., 2008)	• Old + vehicle:<br>○ Decrease CB1 receptor enhanced glutamate release.<br>○ Increase excitotoxicity to the neuron.<br>○ Promoting glial cell reactivity.	• Old + WIN 2:<br>○ restore a proper calcium influx via NMDA channels in a manner similar to that described for the NMDA.<br>○ stimulates CB receptors on hippocampal neurons to modulate glutamatergic and GABAergic function.<br>○ Reduced microglial activation.
(Ramírez et al., 2005)	• Mice Aβ intracerebroventricularly injected of:<br>○ CB1 receptor protein expression markedly decreased in AD brains.<br>○ CB1 and CB2 proteins show enhanced nitration.<br>○ Nitration of CB1 and CB2 protein was markedly increased.<br>○ increased microglial reaction.	• Mice intracerebroventricularly injected treated with WIN55:<br>○ Attenuation of neuronal loss markers induced by Aβ.<br>○ Reduce microglial activation and decrease NO production.<br>○ Decrease TNFα release.
